# Electrochemical and Kinetic Insights into Molecular Water Oxidation Catalysts Derived from Cp*Ir(pyridine‐alkoxide) Complexes

**DOI:** 10.1002/cctc.201800916

**Published:** 2018-09-30

**Authors:** Emma V. Sackville, Frank Marken, Ulrich Hintermair

**Affiliations:** ^1^ Centre for Sustainable Chemical Technologies University of Bath Claverton DownBath BA2 7AY United Kingdom; ^2^ Department of Chemistry University of Bath Claverton Down Bath BA2 7AY United Kingdom

**Keywords:** Iridium complexes, pyridine-alkoxide ligands, homogeneous catalysis, electrochemistry, water oxidation

## Abstract

We report the solution‐phase electrochemistry of seven half‐sandwich iridium(III) complexes with varying pyridine‐alkoxide ligands to quantify electronic ligand effects that translate to their activity in catalytic water oxidation. Our results unify some previously reported electrochemical data of Cp*Ir complexes by showing how the solution speciation determines the electrochemical response: cationic complexes show over 1 V higher redox potentials that their neutral forms in a distinct demonstration of charge accumulation effects relevant to water oxidation. Building on previous work that analysed the activation behaviour of our pyalk‐ligated Cp*Ir complexes **1**–**7**, we assess their catalytic oxygen evolution activity with sodium periodate (NaIO_4_) and ceric ammonium nitrate (CAN) in water and aqueous ^t^BuOH solution. Mechanistic studies including H/D kinetic isotope effects and reaction progress kinetic analysis (RPKA) of oxygen evolution point to a dimer‐monomer equilibrium of the Ir^IV^ resting state preceding a proton‐coupled electron transfer (PCET) in the turnover‐limiting step (TLS). Finally, true electrochemically driven water oxidation is demonstrated for all catalysts, revealing surprising trends in activity that do not correlate with those obtained using chemical oxidants.

## Introduction

The conversion and storage of renewable electricity from wind, tidal and solar power in chemical fuels is a promising strategy to overcome their inherent drawbacks of diffusivity and intermittency. The oxidation of water could provide the reducing equivalents needed for the production of zero carbon fuels on large scale, but the kinetic challenges of the water oxidation half reaction constitutes a major bottleneck in the realisation of this scenario. Efficient and robust water oxidation catalysts (WOCs) may reduce losses and speed up conversion rates to help make renewable energy more widely usable.

A wide range of WOCs have been reported, both heterogeneous^1^ and homogeneous,^2^ mainly based around Mn,^3^, ^4^, ^5^, ^6^ Ru^7^,^8^ and Ir^9^ as the active metal. Although heterogeneous WOCs are often easier to fabricate and said to be more robust, molecular WOCs offer higher atom economy and are exciting from the view of mechanistic understanding and the possibility of fine‐tuning the active site. Mononuclear iridium catalysts in particular have come to the fore since the first report by Bernhard and co‐workers 10 years ago.^10^ Since then a wide number of molecular iridium precursors have been reported, with half sandwich iridium compounds showing the highest activities.^9^,^11^, ^12^, ^13^, ^14^ Although the exact nature of the active species is still a matter of debate, it has been shown that the Cp*Ir^III^ complexes are precursors which undergo oxidative activation with loss of the Cp* ligand,^15^, ^16^, ^17^ either chemically^18^,^19^ or electrochemically,^20^ before entering catalysis.^21^, ^22^, ^23^ A crucial feature of the most effective members of that family is an oxidatively robust chelate ligand that remains bound to the iridium to prevent decomposition into IrO_x_ and modulates the active site.^24^,^25^ Pyridine‐alkoxides have emerged as privileged ligands in this chemistry due to their combination of high donor power and oxidative resilience.^27^

**Figure 1:**
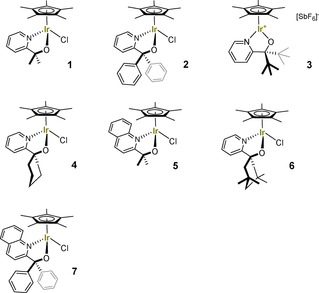
Cp*Ir^III^ pyridine‐alkoxide precatalysts **1**–**7** investigated for water oxidation.

We have recently reported the synthesis of a series of pyridine‐alkoxide and quinoline‐alkoxide (collectively abbreviated as ‘pyalk’) ligated Cp*Ir^III^ complexes **1**–**7** (Figure 1), and have shown how the ligand substitution pattern affected the solution speciation, pre‐catalytic activation, and catalytic C−H oxygenation with aqueous NaIO_4_.^23^,^26^ It was found that under typical reaction conditions (μM to mM [Ir] concentrations in neutral aqueous solution at room temperature), all complexes **1**–**7** readily dissociated the halide ligand to become available for oxidative activation by hydrated periodate, a process which was fast relative to C−H oxidation under catalytic conditions. Thus, all ligand effects observed within the series originated from catalytic turnover, substantiating the notion the ligands remain bound to the active site after activation in each case. Monitoring oxygen evolution during C−H oxidation catalysis showed these two competing reactions to occur sequentially, with the more active catalysts bearing ligands of higher donicity and lower steric bulk diverting more of the oxidant towards the initial O_2_ evolution reaction (seconds to minutes) before C−H oxidation took place (minutes to hours). Herein we now focus on their electrochemical behaviour and mechanistic details of catalytic oxygen evolution.

## Results and Discussion

We began our investigation into the electrocatalytic activity of these catalysts by studying the solution electrochemistry of the precursor complexes **1**–**7**. Cyclic voltammetry (CV) data of several Cp*Ir based oxidation catalysts have been published,^11^,^13^,^20^,^27^, ^28^, ^29^, ^30^, ^31^ but a clear assignment of the different redox events reported under the various conditions applied is still lacking. For instance, the observation of a catalytic wave at 1.4–1.6 V vs. NHE in aqueous solution initially ascribed to the onset of catalytic water oxidation^27^,^29^, ^30^, ^31^ has later been shown to originate from incipient precursor activation by C−H oxidation of the Cp* ligand.^20^ Sometimes the CVs contain signatures of different species formed *in‐situ* under the potentials applied, which may be assigned incorrectly unless careful control experiments are performed. For instance, quasi‐reversible pre‐catalytic features around 0.9 V vs. NHE often assigned to a molecular Ir^III–IV^ redox couple^30^ are more characteristic of amorphous, hydrated iridium‐oxyhydroxide deposites^32^, ^33^, ^34^, ^35^ which may form on the surface of the working electrode.^36^, ^37^, ^38^ The recent electrochemical characterization of well‐defined and stable model complexes showing Ir^III–IV^ redox couples at potentials below 0.7 V vs. NHE and even reversible Ir^IV–V^ transitions around 1.0–1.2 V vs. NHE^39^,^40^ call for a revision of the electrochemistry of Cp*Ir based water oxidation precatalysts.

**Figure 2:**
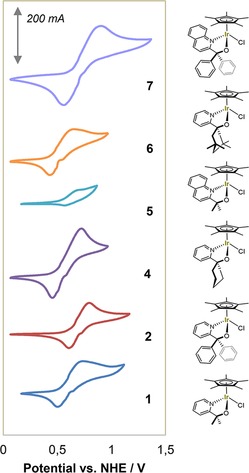
Cyclic voltammograms of complexes **1**, **2**, **4**–**7** at 10 mM [Ir] in dry CH_2_Cl_2_ with 0.15 M [tmbim][NTf_2_] electrolyte under Argon at room temperature (WE: 3 mm glassy carbon disc, RE: Ag/AgNO_3_, CE: 1 mm Pt wire, SR: 100 mV s^−1^).

Initially, our cyclic voltammograms collected for complexes **1**–**7** in aqueous media were complicated by partial oxidation of the easily activated precatalysts when scanning to positive potentials. All attempts to suppress this by variation of electrode materials, scan rates or electrolyte were unsuccessful, and multiple redox features originating from several species were always observed (Figure S1). In addition, when using working electrode materials that consisted of or formed oxide layers during the experiment, surface binding of the activated catalyst species occurred during the experiment as shown by control experiments (Figure S1). This reactivity may be beneficially exploited for grafting these catalysts onto conducting metal oxides to furnish highly efficient and robust water‐oxidation anodes,^14^ but in this case added to the challenge of analysing the electrochemistry of the precursor complexes in solution. Only by conducting the cyclic voltammetry in the strict absence of water and oxygen inside an argon‐filled glovebox, meaningful electrochemical data for **1**–**7** could be obtained. Using thoroughly cleaned glassy carbon working electrodes with an oxidatively stable ionic liquid – type electrolyte ([tmbim][NTf_2_], see supporting information) in a non‐coordinating solvent (dry methylene chloride), we reproducibly obtained clean CVs for complexes **1**–**7** without any signs of solution phase decomposition or deposition on the electrode surfaces (Figures 2 and S2).

All complexes except **3** showed quasi‐reversible one electron transfer events between 0.55 and 0.75 V vs. NHE, which in the absence of any further chemical transformations can now unambiguously be assigned to the Ir^III–IV^ redox couple (Figure S3). No degradation was observed during extended potential cycling and variation of scan rates, testament to the stability of pyalk‐type ligands in higher oxidation state complexes.^24^ The high resistivity of the solvent under inert conditions meant that peak separations of the anodic and cathodic events were far from the ideal 59 mV even for ferrocene (Figure S3), but thermodynamic mid‐point potentials *E_mid_* could be extracted from the CVs at varying scan rates (Figure S4).

**Figure 3:**
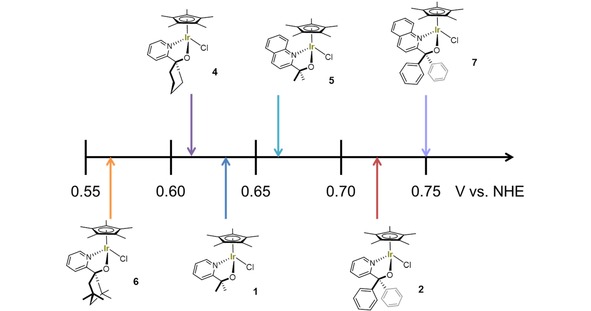
Mid‐point potentials of the Ir^III–IV^ redox couple for complexes **1**, **2**, **4**–**7** as obtained from cyclic voltammetry (Figures 2 and S4).

The *E_mid_* values of the pyridine‐alkoxide ligated Cp*Ir^III–IV^ in **1**–**7** varied by almost 200 mV as a function of the ligand substitution pattern (Figure 3, Table S1). The alkyl‐substituted complexes all fell in the 0.56–0.66 V vs. NHE region, whereas the aryl‐substituted complexes exhibited ∼100 mV higher redox potentials. Extending the pyridine backbone to a quinoline system added another 30 mV. This is consistent with our previous finding of **2** and **7** acting as precursors to slower but more C−H selective oxidation catalysts compared to the alkyl‐substituted pyridine alkoxide complexes.^26^ Therefore, cyclic voltammetry (under appropriate conditions) provides a mean of quantifying these electronic ligand effects in the precursor complexes.

**Figure 4:**
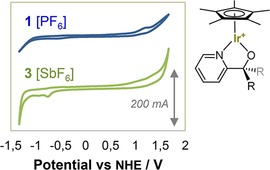
Cyclic voltammograms of complexes **1**[PF_6_] and **3**[SbF_6_] at 10 mM [Ir] in dry CH_2_Cl_2_ with 0.15 M [tmbim][NTf_2_] electrolyte under Argon at room temperature (WE: 3 mm glassy carbon disc, RE: Ag/AgNO_3_, CE: 1 mm Pt wire, SR: 100 mV s^−1^).

Interestingly, the cationic complex **3** showed no redox features up to 1.5 V vs. NHE. This suggested a marked difference in the electrochemical behaviour of the neutral, six‐coordinate chloride complexes *versus* the cationic, five‐coordinate [Cp*Ir($N^{\wedge }O$ )]^+^ complex form. Indeed, when **1** was converted into its cationic form by halide abstraction with NaPF_6_,^41^ the reversible features around 0.6 V vs. NHE observed for the neutral, octahedral chloride complex **1** completely disappeared (Figure 4).

Although the HOMO (*d_xy_* for low‐spin d^6^) in a distorted trigonal bipyramidal coordination geometry is higher in energy than the HOMO of an octahedral complex (t_2g_ for low‐spin d^6^),^42^ the electrostatic effect of a net positive charge apparently raises the Ir^III–IV^ couple by >1 V. This is consistent with the electrochemistry reported for the six‐coordinate cationic complex [Cp*Ir(phenylpyridine)MeCN]^+^ in acetonitrile, which showed no redox features until an irreversible oxidation peak at 1.6 V vs. NHE.^43^ The need for minimising charge accumulation during the 4‐electron water oxidation cycle by (stepwise or coupled) proton transfer events^44^ or distribution over several metal centres^45^ in order to level the redox potentials throughout the catalytic cycle is well known,^46^ and a direct observation of the effect of charge accumulation on these widely studied Cp*Ir precatalysts provides a measurable basis for further catalyst fine‐tuning and ligand design.

### Kinetics of Chemical Water Oxidation

**Figure 5:**
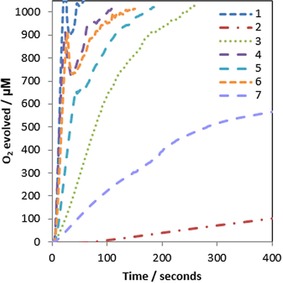
Oxygen evolution traces of precatalysts **1**–**7** at 100 μM [Ir] with 100 mM NaIO_4_ in H_2_O (native pH 5.6) at 25 °C using a calibrated Clark electrode with stirring (dents caused by O_2_ bubble formation).

Previously we have monitored O_2_ evolution during NaIO_4_‐driven catalytic C−H oxidation of ethylbenzene‐sulfonate (EBS) in ^t^BuOH/H_2_O mixtures with **1**–**7** to establish a correlation between activity and selectivity of the different catalysts.^26^ Here we now report the kinetics of pure O_2_ evolution activity with NaIO_4_ in neat aqueous solution, measured with a Clarke‐type electrode in the liquid phase (Figure 5).

In neat water, O_2_ evolution activity was about one order of magnitude higher than in the presence of oxidizable organic substrates for all complexes (Table 1). Precursor **6** (with the lowest *E_mid_* of all) showed the most dramatic activity increase of a factor of 45, while precursor **7** (with the highest *E_mid_* of all) only increased its activity two‐fold as compared to the presence of EBS. This observation shows the electronic ligand effects observed in the precursor complexes by cyclic voltammetry (Figures 2 & 3) to translate into their active species, rendering **6** the easiest and **7** the (electronically) hardest catalyst to be turned over by NaIO_4_. The fact that **4** and **1** surpass the activity of **6** despite their slightly higher Ir^III–IV^ potentials is likely a reflection of more favourable exchange kinetics (oxidant, water, protons) due to reduced steric bulk.

**Table 1:** Initial rates of oxygen evolution of precatalysts **1**–**7** with NaIO_4_ and CAN from Figures 5 and 6.

Precatalyst	initial *k_obs_* ^[a]^ with NaIO_4_ [mM min^−1^ **]**	catalyst TOF^[b]^ with NaIO_4_ [h^−1^]	initial *k_obs_* ^[a]^ with CAN [mM min^−1^]	catalyst TOF^[b]^ with CAN [h^−1^]
**1**	4.42±0.053	2739±32	1.84±0.126	1105±74
**2**	0.02±0.001	11±0.6	0.22±0.050	133±30
**3**	0.42±0.015	248±9	0.45±0.049	270±29
**4**	3.71±0.020	2167±11	1.88±0.185	1128±110
**5**	1.23±0.057	738±34	1.22±0.054	732±32
**6**	2.88±0.029	1728±17	1.12±0.072	672±43
**7**	0.14±0.008	83±5	0.09±0.004	54±2

[a] Calculated from the initial gradient of O_2_ formation over time as the average from triplicates (see Table S2); [b] Initial rate divided by [Ir] concentration. Errors calculated from standard deviation of rate from repeat runs.

**Figure 6:**
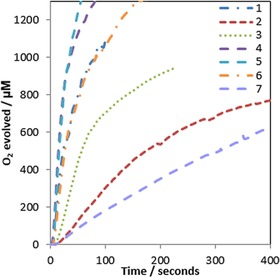
Oxygen evolution traces of precatalysts **1**–**7** at 100 μM [Ir] with 200 mM CAN in 0.1 M HNO_3_ in H_2_O (pH 1.5) at 25 °C using a calibrated Clark electrode with stirring (dents caused by O_2_ bubble formation).

The water oxidation activities of **1**–**7** were also assessed with ceric ammonium nitrate (CAN) as a stronger one‐electron oxidant at lower pH.^47^ All precatalysts proved active (Figure 6); the faster catalysts **1**, **4**, and **6** were less effective with CAN than with NaIO_4_ (2–3 times lower rate), whereas the slower ones showed about the same level of activity. Only complex **2**, barely active with NaIO_4_, exhibited markedly higher activity (∼10 times faster) with the stronger oxidant CAN. The fact that **7** (with an even higher Ir^III–IV^ *E_mid_*) showed lower rates with CAN than with NaIO_4_ might be a reflection of partial deligation facilitated by the strongly acidic media.

We also briefly investigated the effect of adding an oxidation‐resistant organic co‐solvent as typically required for catalytic C−H oxidations with these catalysts.^16^ Previously we found adding 20 vol % *tert*‐butanol to be most efficient for this purpose,^26^ so O_2_ evolution assays with NaIO_4_ were repeated in 4 : 1 H_2_O/^t^BuOH (Figure S5) but without any organic substrate present. We were surprised to find significant rate reductions in O_2_ evolution (3–4 times slower) for the faster catalysts caused by the presence of 20 % ^t^BuOH (Table 2). Only the two slowest catalysts derived from **2** and **7** did not experience much change. While at present we can't offer a rationale for this effect yet, it is clear that one the reason for the higher C−H oxidation efficiencies obtained in aqueous ^t^BuOH is that the co‐solvent steers the C−H vs. O−H oxidation competition more towards C−H oxidation by channelling less oxidant into the O_2_ evolution cycle with the more active catalysts. Catalysts which are inherently slower are less influenced by this solvent effect, as previously shown by **2** as the most efficient C−H oxidation catalyst of the series.^26^

**Table 2:** Initial rates of oxygen evolution of precatalysts **1−7** with NaIO_4_ in pure water and with 20 vol % ^t^BuOH added (Figures 5 and S4).

Precatalyst	initial *k_obs_* ^[a]^ in pure H_2_O [mM min^−1^]	initial *k_obs_* ^[a]^ in 4 : 1 H_2_O/^t^BuOH [mM min^−1^]	rate reduction by ^t^BuOH
**1**	4.42±0.053	1.26±0.008	73 %
**2**	0.02±0.001	0.02±0.002	0 %
**3**	0.42±0.015	0.15±0.025	62 %
**4**	3.71±0.020	1.25±0.026	65 %
**5**	1.23±0.057	0.18±0.022	75 %
**6**	2.88±0.029	0.91±0.076	74 %
**7**	0.14±0.008	0.13±0.007	10 %

[a] Calculated from the initial gradient of O_2_ formation over time as the average from triplicates (see Table S2). Errors calculated from standard deviation of rate from repeat runs.

As a way of gaining some mechanistic insight into how these catalysts operate, H/D kinetic isotope effects (KIE) for O_2_ evolution from aqueous NaIO_4_ were measured (Figure S6). As we have already seen multiple pieces of evidence for ligand effects on turnover in the series of **1**–**7**, it was interesting to test how similar the active sites in the catalysts derived from **1**–**7** may be, and whether they go through the same turnover limiting step (TLS). Under non‐competitive conditions, all catalysts showed a positive or normal H/D KIE>1 indicative of O−H bond breaking to be part of the TLS that is slower in case of O−D. Consistent with previous literature^20^ **1** showed a KIE of 2.1, and values ranging from 1.3 up to 2.5 were obtained for **2**–**7** at 25 °C (Table 3). If we assume the absence of equilibrium and solvation isotope effects (which is reasonable given that the solvent is the substrate that binds and exchanges rapidly with both the oxidant and the catalyst), all of these values are in the range of primary KIEs indicative of O−H cleavage to be directly involved in the TLS.^48^ Since the Ir^IV^ level of the activated catalysts is known to be a stable resting state (as shown for **1** 20), we propose that concerted PCET to a higher oxidation state intermediate such as an Ir^V^ oxo is the TLS of the catalytic cycle (see also further below). The different electronics and H‐bonding capabilities of the variously substituted pyalk‐type ligands in **1**–**7** thus each give rise to different barriers for this rate‐determining step. Also, all are distinct from aq. IrO_x_ nanoparticles, which operate via a mechanism where O−H cleavage is not turnover limiting as shown by the absence of a measurable H/D KIE (i. e. rate ratio of 1.0).^49^

**Table 3:** Kinetic H/D isotope effects of oxygen evolution with precatalysts **1**–**7** and NaIO_4_ in pure water at 25 °C.

Precatalyst	initial *k_obs_* ^[a]^ in H_2_O [mM min^−1^]	initial *k_obs_* ^[a]^ in D_2_O [mM min^−1^]	H/D KIE ^[b]^
**1**	4.42±0.053	2.14±0.107	2.15±0.17
**2**	0.02±0.001	7.2×10^−3^±7.7×10^−4^	2.50±0.34
**3**	0.42±0.015	0.32±0.010	1.27±0.07
**4**	3.71±0.020	1.88±0.114	1.92±0.16
**5**	1.23±0.057	0.75±0.061	1.73±0.19
**6**	2.88±0.029	1.46±0.143	1.96±0.18
**7**	0.14±0.008	0.11±0.011	1.26±0.16

[a] Calculated from the initial gradient of O_2_ formation over time as the average from triplicates (see Table S2); [b] Calculated as initial k_obs_ in H_2_O/initial k_obs_ in D_2_O. KIE errors calculated from standard deviation of upper and lower limits of rates.

In order to obtain further mechanistic insight we sought to investigate the kinetics of the O_2_ evolution reaction with **1**–**7**. Kinetic data of Cp*Ir‐based water oxidation precatalysts using initial rate analyses have been published,^27^ but these mostly provide information on the precatalytic activation step which can be expected to follow a different rate law than the ensuing catalytic turnover. Reek has recently applied reaction progress kinetic analysis (RPKA)^50^ to a series of Cp*Ir based WOCs by following CAN consumption via UV‐vis spectroscopy.^51^ Fractional orders in precursor and changes in rate behaviour over time were observed for all complexes tested, plausibly because the analysis was based on the rate of disappearance of oxidant that is consumed in both the activation step and catalytic turnover. In addition, CAN and its reduced forms are known to interfere with the water oxidation cycle by engaging in oxygen‐exchange mechanisms^52^ and forming ceria nanoparticles, which have been reported to induce catalyst degradation and cause heterogeneous background activity in oxygen evolution.^21^ Lastly, the strongly acidic media required for using Ce^4+^ as sacrificial oxidant for water oxidation (pH∼1) may lead to modification of some of the precursors even before addition of the oxidant (as in our own observations when using **7** with CAN; see Table 1). Thus, we decided to use NaIO_4_ as mild, pH neutral and fully homogeneous oxidant,^53^ and based our reaction progress kinetic analysis on the catalytic formation of oxygen over time as detected by a Clarke‐type electrode. This way we did not include any data from non‐productive precursor activation, and avoid exogenously induced catalyst decomposition and background activity. We apply Burés’ variable time normalization analysis (VTNA) method for graphical analysis of reaction orders directly from product formation profiles,^41^ but note that Blackmond's original graphical rate equations would yield the same results after differentiation of the data.^50^ Highlighting the importance of identifying appropriate concentration regimes for kinetic analyses, we initially found the system to be zeroth order in [Ir] throughout the entire reaction profile under standard reaction conditions (Figure S7). By iteratively changing [Ir] and oxidant concentrations, the system could be brought out of the saturation regime to converge to catalyst orders of ∼0.5 for precursor **1** (Figure S8 and Table 4). 
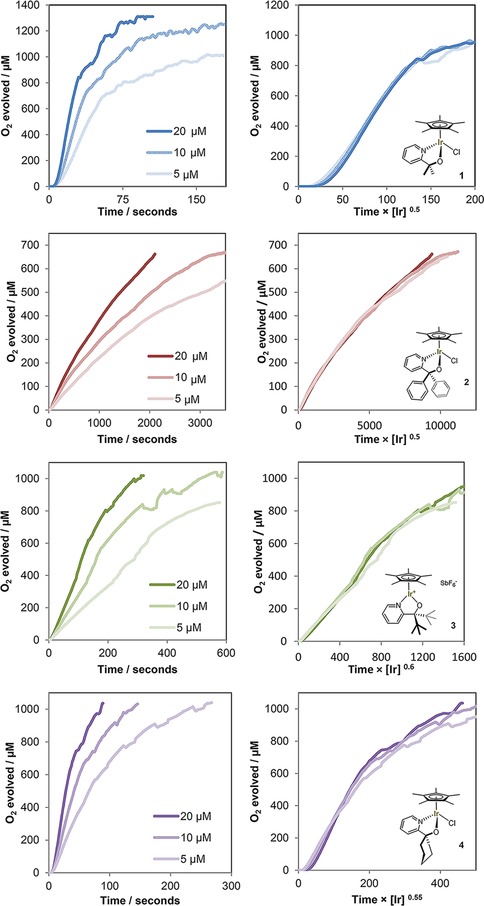


**Table 4:** Order in [Ir] for catalytic O_2_ evolution with **1** and NaIO_4_ in different concentration regimes (data collected as in Figure 5).

Concentration of NaIO_4_ [mmol L^−1^]	Concentration of [1] [μmol L^−1^]	Order in [Ir] from VTNA
10	200 – 100 – 50	0
100	200 – 100 – 50	0.3
10	10 – 5 – 2.5	0.5
100	20 – 10 – 5	0.5

**Figure 7:**
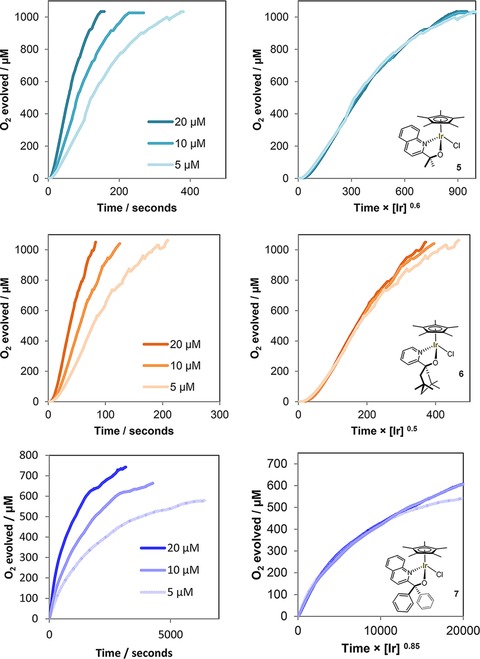
Oxygen evolution traces of precatalysts **1**–**7** at various [Ir] concentrations with 100 mM NaIO_4_ in H_2_O (native pH 5.6) at 25 °C using a calibrated Clark electrode with stirring as measured (**left**; dents caused by O_2_ bubble formation), and with variable time normalization applied (**right**).

Reaction kinetics with different catalyst loadings were then investigated for all precursors by VTNA of their O_2_ formation profiles under these optimised conditions. Figure 7 shows the best fits in iridium order for each complex as the power of the concentration factor in the normalized time axes (for alternative fitting attempts see Figure S9).

It is striking that catalysts **1**–**6** gave very good fits for [Ir] orders of 0.5–0.6 throughout the reaction. In some cases there were minor deviations at longer reaction times that may be taken as signs of deactivation, but we note that Clark electrode measurements become less accurate at higher O_2_ contents and longer reaction times due to oxygen escaping the analysis by diffusing out of the chamber. We conclude that the fact that one reaction order overlaid all profiles in their entirety (within the accuracy of the experiment) suggests that there are no significant changes in the mechanism throughout the reaction. No rate accelerations indicative of nanoparticle formation were seen either with any of the precursors tested. In their UV‐vis RPKA study with CAN, Reek have found [Ir] orders of around 1.7 for **1** and related compounds at pH 1,^51^ however, without taking the 4 : 1 stoichiometry of oxidant to product into account. Initial rate analysis of [Cp*Ir(NHC)(OH)_2_] with NaIO_4_ using a Clarke‐type electrode detecting O_2_ reportedly gave a 0.65 order in [Ir];^54^ close to our findings of 0.5–0.6. In pH 7 phosphate buffer, [Ir] orders of 0.85–0.98 have been reported for a selection of different Ir‐based WOCs with aqueous NaIO_4_, however, the analysis was performed at the “point of maximum rate” and not via RPKA of the full reaction profiles.^55^

**Figure 8:**
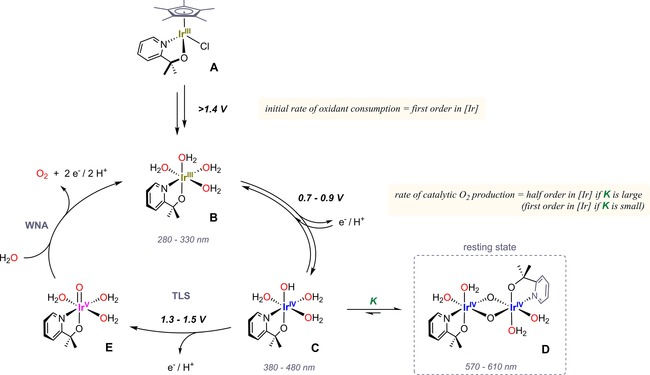
Expanded catalytic cycle for water oxidation starting from pyalk‐ligated Cp*Ir^III^ precursor complexes (using **1** as an example) with redox potential ranges (vs. NHE) and characteristic UV‐vis absorptions of key intermediates (net charges and protonation states will depend on pH).

An order <1 in iridium for the rate of O_2_ formation is particularly interesting as it suggests the existence of dimeric species breaking up into active monomers that generate product in the TLS.^56^ XPS, EPR, resonance‐Raman, ^17^O‐NMR and other techniques have previously established the resting state of activated **1** to be an oxo‐bridged Ir^IV^ dimer,^23^ and a range of dinuclear model complexes bearing the same ligand have recently been synthesized.^39^,^57^, ^58^, ^59^, ^60^, ^61^, ^62^, ^63^ The kinetic relevance of these dimers had not been elucidated yet, however. A half order in [Ir] on the rate of O_2_ evolution would imply these dimers dominate the solution speciation of the catalyst under turnover conditions, but liberate small amounts of active monomers into the productive cycle (Figure 8).^56^ This scenario is consistent with the reported high stability of *μ*‐oxo Ir complexes,^64^,^65^ and the observation that only minor colour changes occur in the UV‐vis during the O_2_ evolution reaction.^23^

This expanded mechanism merges the identification of stable dimeric resting states with the previously postulated mono‐nuclear pathway proceeding through an Ir^III–IV–V^ sequence.^27^ By providing kinetic evidence for monomeric active sites it further disfavours bimetallic oxo coupling pathways^11^ and lends additional support to a water nucleophilic attack (WNA) mechanism on an Ir^V^ oxo to furnish the O−O bond.^43^ While the exact geometries, coordination numbers and protonation states of species **B**, **C**, **D** and **E** in Figure 8 remain to be ascertained, this simple scheme does explain a number of key features of this chemistry. Activation of the electronically and coordinatively saturated pre‐catalyst **A** has a relatively high redox barrier for the initial Cp* hydroxylations to occur,^15^ but once overcome is irreversible and leads directly into the catalytic cycle throughout which the pyalk ligand is retained. This step dominates the aqueous electrochemistry of the precursor^20^ as well as the initial rate of oxidant consumption with the expected first order in [Cp*Ir].^23^ Once the solution potential is exhausted, the resting state of the activated catalyst is the blue Ir^IV^ dimer **D**, which can be reduced to the yellow Ir^III^ complex **B**. This reversible interconversion may in principle involve a dimeric version of **B**, although a coordinatively and electronically saturated octahedral Ir^III^ would have little driving force for dimerization. The persistent dimeric nature of the Ir^IV^ **D** on the other hand explains why no EPR signatures can be obtained for the *d* ^*5*^ centres (antiferromagnetic coupling),^23^ and why their characteristic blue colour persists throughout the reaction. The formulation of proton‐coupled oxidation of **C** to **E** as the most reactive species involved in the TLS is consistent with our findings of half order in [Ir] and significant primary H/D KIE values. The fact that **7** gave an order of 0.85 suggests that in this case the dimer‐monomer equilibrium *K* lies more towards the monomer (a less active one due to electronic reasons), which is further consistent with its resting state showing low intensity around 600 nm^26^ where the characteristic dπ–pπ* transitions of the Ir^IV^‐O‐Ir^IV^ unit occur.^23^

### Electrochemical Water Oxidation

Chemical oxidants are convenient for catalyst development and benchmarking as the kinetics can easily be measured, but they are not reliable indicators for true electrocatalytic oxygen evolution due to different e‐transfer pathways, varying solution potential throughout the reaction, and possible chemical interference.^66^ Thus, if any WOC is to be useful for renewable energy conversion by applied water splitting, its true electrocatalytic behaviour must be assessed. Solid‐state anodes with spatially and temporarily fixed active centres on the electrode surface are easily characterised by measuring overpotentials, but the situation is more complicated for freely diffusing solution‐phase species, where the amount of catalyst contributing to the current measured is unknown.^67^ One reason for the limited number of electrochemical oxygen evolution data reported in the literature are the difficulties in quantitatively interpreting voltammograms of homogeneous electrocatalysts. Several electrochemical methods for estimating the amount of solution‐phase catalyst contributing to the current measured to extract their intrinsic rate constant have been described,^67^, ^68^, ^69^ but very few apply to water oxidation in aqueous solution where substrate‐limited plateau currents are not achievable. Foot‐of‐the‐wave analysis (FOWA) has recently been applied to WOCs as a tool to extract rate constants at the onset of the electrocatalysis by correlating the catalytic current with that of another reversible (pre‐catalytic) redox feature of the catalyst.^69^,^70^ Although these values often greatly over‐estimate the true performance of the catalyst at higher potentials as required for practical application, FOWA is a useful tool for evaluating and comparing performance during molecular electrocatalyst development.

Initially, when aqueous CV data on complexes **1**–**7** preactivated with 50 equivalents of NaIO_4_ were measured with NaNO_3_ as the electrolyte, we observed very similar features at positive potentials for all solutions with a variety of working electrode materials (Figure S10). All samples showed an irreversible oxidation peak around 1.6 V vs. RHE and the onset of a broad catalytic wave around 1.8 V vs. RHE. Control experiments revealed these to originate mostly from NaIO_3_, the reduced form of the oxidant required for precursor activation (Figure S10). There were some differences in the CVs from underlying catalyst contributions, but the necessity of using an excess of chemical oxidant for quantitative precursor activation, and the absence of any clearly defined pre‐catalytic redox feature of the catalyst obscured evaluation of electrocatalytic performance purely by electrochemical techniques.

**Figure 9:**
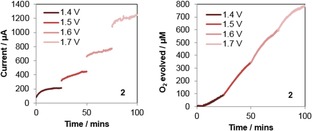
Chronoamperometry (**left**) and oxygen evolution (**right**) traces for electrochemically driven water oxidation using complex **2** pre‐activated with 100 equivalents of NaIO_4_ for 24 hours prior to the experiment (2.5 mM [Ir], 250 mM NaIO_3_, pH 6, 25 °C with stirring, WE: 0.25 cm^2^ BDD plate, CE: 1 mm Pt wire, RE: Ag/AgCl).

We thus opted for a direct detection approach, where the working electrodes were inserted into a stirred chamber above an independent Clark electrode (see supporting information 3.1 for details). As not all oxygen generated was effectively transported to the point of detection we could not quantify Faradaic efficiencies or overpotentials this way, but having an independent and selective way of detecting product *in‐situ* from electrochemically driven water oxidation is an unbiased method of testing the true water oxidation ability of a molecular WOC. After optimisation of electrode materials and positioning (see supporting information 3.2 for details) we reproducibly obtained O_2_ responses that tracked the current flow during chronoamperometry at different potentials. The use of a boron‐doped diamond (BDD) electrode as stable, non‐catalytic working electrode material^71^,^72^ was key to eliminating background activity and catalyst decomposition as shown by negative blank tests after each experiment (Figure S16). Figure 9 exemplifies the results obtained for activated **2**, and the data of all other catalysts can be found in the supporting information (Figure S15).

**Figure 10:**
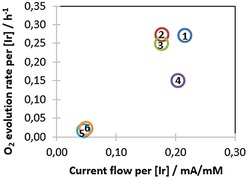
O_2_ evolution rate (Clark electrode) over current flow (potentiostat) of **1**–**7** pre‐activated with 100 equivalents of NaIO_4_ for 24 hours prior to the experiment (2.5 mM [Ir], 250 mM NaIO_3_, pH 6, 25 °C with stirring, WE: 0.25 cm^2^ BDD plate, CE: 1 mm Pt wire, RE: Ag/AgCl) at 1.5 V vs. NHE applied potential.

Although the amount of O_2_ produced was transport‐limited at potentials above 1.5 V vs. RHE (corresponding to a maximum detectable rate of 50 μM/min), all catalysts showed different responses in their initial current flow and O_2_ production (Figure S15). Strikingly, at 1.5 V vs. NHE the order of activity in electrochemical O_2_ evolution was quite different to reactivity seen before with NaIO_4_ and CAN: while **5**, **6**, and **7** were essentially inactive (minor current flow but no detectable O_2_ production) **1**, **2**, **3**, and **4** showed good activity. Plotting O_2_ evolution vs. current summarises the collective results obtained (Figure 10).

While with chemical oxidants **6** was the third most active oxygen evolution catalyst of the series, under electrochemical conditions it turned out to be essentially inactive (as was **5**). **1** had thus far been found to excel under all conditions applied, but the observation that **2** rivals the performance of **1** under electrocatalytic conditions is remarkable; its precursor complex **A** has one of the highest Ir^III–IV^ redox potentials (0.72 V vs. NHE), shows the lowest λ_max_ in the activated form **D** (567 nm), gives the highest C−H oxidation efficiency in aqueous NaIO_4_ with zero co‐generation of O_2_, and has one of the lowest O_2_ activities with both chemical oxidants but the highest H/D KIE of 2.5. It appears that under the influence of a steady catalytic potential supplied by an electrode its active monomer **C** is either more efficient at accumulating charges and turning over water to oxygen (due to electronic effects of the diphenyl‐substituted ligand), or that its equilibrium *K* lies more towards **C** than when using chemical oxidants. This question and how surface‐binding of these catalysts affects the situation remain to be answered in future studies, but these findings illustrate again how mechanisms may shift depending on the conditions applied^66^ and that true electrochemical oxygen evolution activity (in conjunction with other methods) must be assessed in order to find the best WOC.

## Conclusions

The pyridine‐alkoxide Cp*Ir complexes **1**–**7** have been shown to be potent water oxidation catalysts under a variety of conditions, exhibiting clear ligand effects from the substitution pattern of the pyalk ligands. In non‐coordinating, anhydrous solvents these are assessable by cyclic voltammetry, revealing the effect of charge accumulation on the redox potentials of the Ir^III–IV^ couple. Primary H/D kinetic isotope effects in the range of 1.3–2.5 provide additional evidence for the retention of the ligands during turnover, and point to O−H cleavage being part of the TLS of the catalytic cycle, plausibly in a PCET step. Analysis of the kinetics of oxygen evolution with NaIO_4_ by RPKA using VTNA showed product formation to be half order in [Ir], consistent with a monomer‐dimer equilibrium of the Ir^IV^ resting state that explains a number of kinetic and spectroscopic features previously observed by us and others.^23^,^30^,^51^ All catalysts have been assessed in electrochemically driven water oxidation, revealing a different order of reactivity topped by the pyridine‐diphenylalkoxide catalyst **2** as the most efficient electrocatalyst. These findings highlight the importance of assessing and validating WOC performance electrochemically, and give valuable clues for future improvement by ligand design. Further analysis of the geometric and electronic structure of their intermediates, both experimentally and computationally, can be expected to afford new exciting prospects for designing improved molecular WOCs for application in renewable energy conversion.

## **Experimental**

All chemicals were purchased from major commercial suppliers and used as received. Triply filtered Milli‐Q water (18 MΩ•cm) was used in all experiments. Catalysts and ligands were synthesised according to previously published procedures.^26^ Generally, [Cp*IrCl_2_]_2_ (0.1 mmol, 79.8 mg), ligand (0.2 mmol), and Na_2_CO_3_ (0.8 mmol, 84.8 mg) were dissolved in dry acetone (15 mL). The resulting orange solution was stirred for 6 h at 50 °C, after which time the solution had turned yellow. MgSO_4_ was added, and after stirring for 10 min the solution was filtered and the solvent removed *in vacuo* to afford an orange‐red solid. The product was recrystallized from DCM by the addition of diethyl ether, the supernatant removed and the powder dried *in vacuo* to give yellow‐orange microcrystals in yields of 54–81 %.

### Electrochemistry

Electrochemical experiments were performed using three‐electrode measurements carried out on an Invium Technologies CompactStat. All non‐aqueous potentials were measured against a Ag/AgNO_3_ reference electrode in acetonitrile (+0.197 V vs NHE^73^), and all aqueous potentials were measured against a Ag/AgCl reference electrode in 3 M KCl, both purchased from Bioanalytical Systems, Inc. The working electrodes used were glassy carbon purchased from Bioanalytical Systems, Inc. (0.3 cm diameter, 0.07 cm^2^ surface area), and counter electrodes were 1 mm diameter platinum wire. Before use, carbon electrodes were thoroughly polished with alumina paste (1.0 μm then 0.3 μm), briefly sonicated (10 seconds), rinsed extensively with Milli‐Q water and dried under a stream of Argon.

*Glove box*: Cyclic voltammograms were collected under inert conditions, with 10 mM [Ir], 0.15 M [tmbIm][NTf_2_] electrolyte in DCM (degassed, freeze pump thawed) at a variety of scan rates. Three scans were collected with the second scan reported.

*Preactivated*: Solutions of 1 mM precatalyst [Ir] were activated with 50 equivalents of NaIO_4_ (50 mM) and 0.1 M NaNO_3_ in 5 mL H_2_O for 24 hours. Cyclic voltammograms were collected using a glassy carbon working electrode and a Pt wire counter electrode as above, with an Ag/AgCl reference electrode.

### Water Oxidation

*In‐situ* oxygen evolution data were collected using a Hansatech Oxygraph Plus system with a DW2/2 Clark‐type electrode chamber (with temperature control and magnetic stirring) measuring dissolved O_2_ in solution. The electrode was prepared with 2 M KCl electrolyte under a PTFE membrane and spacer paper, and the instrument was zeroed with the appropriate background solution depending on the reaction (e. g. 100 mM NaIO_4_ solution in H_2_O or 200 mM CAN solution in H_2_O) thoroughly degassed with argon until stable, minimum O_2_ readings were obtained. Standard conditions were 100 mM NaIO_4_ in H_2_O (2 mL) with the reaction started with the addition of 40 μL of a 5 mM stock solution of the desired [Ir] catalyst in H_2_O giving a final [Ir] concentration of 100 μM. Using Ce^IV^, 200 mM CAN in 0.1 M HNO_3_ in H_2_O (pH 1.5) (2 mL) were used, with the reaction started with the addition of 40 μL of a 5 mM stock solution of the desired [Ir] catalyst in H_2_O, giving a final [Ir] concentration of 100 μM.

*Solvent effects*: 100 mM NaIO_4_ in 20 % ^t^BuOH in H_2_O (2 mL) with the reaction started with the addition of 40 μL of a 5 mM stock solution of the desired [Ir] catalyst in H_2_O/tBuOH (4 : 1), giving a final [Ir] concentration of 100 μM.

*VTNA data*: 100 mM NaIO_4_ in H_2_O (2 mL). Reaction was started by the appropriate addition of [Ir] from 5 mM stock solution. For 50 μM [Ir] final concentration 20 μL of [Ir] stock solution, for 100 μM [Ir] final concentration 40 μL of [Ir] stock solution, for 200 μM [Ir] final concentration 80 μL of [Ir] stock solution.

## Conflict of interest

U.S. Patent 9/790/605 by UH et al. contains intellectual property described in this article.

## Supporting information

As a service to our authors and readers, this journal provides supporting information supplied by the authors. Such materials are peer reviewed and may be re‐organized for online delivery, but are not copy‐edited or typeset. Technical support issues arising from supporting information (other than missing files) should be addressed to the authors.

Supplementary
