# Supplementary material for: Electrochemical and Kinetic Insights into Molecular Water Oxidation Catalysts Derived from Cp*Ir(pyridine‐alkoxide) Complexes
Source: ChemCatChem. 2018 Sep 30;10(19):4280–91. doi: 10.1002/cctc.201800916 (PMC6470865; doi:10.1002/cctc.201800916)
Supplement: Supplementary file 1 — Supplementary [file CCTC-10-4280-s001.pdf]

Supporting Information

© Copyright Wiley-VCH Verlag GmbH & Co. KGaA, 69451 Weinheim, 2018

Electrochemical and Kinetic Insights into Molecular Water Oxidation Catalysts Derived from Cp*Ir(pyridine-alkoxide) Complexes

Emma V. Sackville, Frank Marken, and Ulrich Hintermair*This publication is part of the Young Researchers Series. More information regarding these excellent researchers can be found on the ChemCatChem homepage.

Electrochemical and Kinetic Insights into Molecular Water Oxidation Catalysts Derived from Cp*Ir(pyridine-alkoxide) Complexes

Emma V. Sackville[1], Frank Marken[2], and Ulrich Hintermair[1]*

1. Centre for Sustainable Chemical Technologies, University of Bath, Claverton Down, Bath BA2 7AY, United Kingdom.

2. Department of Chemistry, University of Bath, Claverton Down, Bath BA2 7AY, United Kingdom.

**Corresponding author:* u.hintermair@bath.ac.uk

Supplementary Information

Contents

1. Synthesis 3

General 3

2. Electrochemistry 5

2.1 Aqueous CVs of precatalysts 1-7 5

1.2 Non-Aqueous CVs of ferrocene and precatalysts 1-7 8

1.2.1 Fresh electrolyte solution after each catalyst run 8

1.2.2 Scan rate dependence of non aqueous CVs for Ferrocene and precatalysts 1-7 9

3. Water Oxidation 14

2.1 Water oxidation with 20% tBuOH 100mM $NaIO_4$ 100µM [Ir] 14

2.3 Water Oxidation with 100mM $NaIO_4$ 100µM [Ir] in D_2O 15

2.6 Rate Averages 16

2.6 Reaction Progress Kinetic Analysis by VTNA 17

3 Electrochemically driven water oxidation 19

3.1 Cyclic Voltammograms of activated Iridium Catalysts 19

3.1 Electrochemical water oxidation set up 20

3.2 Electrode optimisation 21

3.4 Catalysts 1,3-7 electrochemcially driven water oxidation 22

3.5 Blank electrolyte between catalyst runs 25

References 27

1. Synthesis

General

Organic solvents were purified by passing over activated alumina with dry argon. All chemicals were purchased from major commercial suppliers and used as received. Syntheses were performed under an inert atmosphere of dry argon using standard Schlenk techniques. NMR spectra were recorded on either 400 or 500 MHz Bruker Avance spectrometers and referenced to residual protio-solvent signals. The chemical shift δ is reported in units of parts per million (ppm).

1,2,3-trimethyl-1H-benzimidazol-3-ium iodide ([tmbim][I])

According to previously published procedures[1], under an inert atmosphere, 2-methyl-1H-benzimidazole (12 mmol, 1.59 g) was dissolved in anhydrous acetonitrile (20 mL) and methyl iodide was added dropwise (15 mmol, 2.13 g, 0.93 mL). After heating at 80 ˚C for 3 days, the resulting mixture was cooled to room temperature and the precipitate filtered, washed with THF and dried in vacuo. A subsequent recrystallisation from ethanol yielded 1,2,3-trimethyl-1H-benzimidazol-3-ium iodide ([tmbim][I]) as a white solid (8.4 mmol, 2.41 g, 70 %).

1,2,3-trimethyl-1H-benzimidazol-3-ium bis((trifluoromethyl)sulfonyl) amide ([tmbim][NTf$_2$])

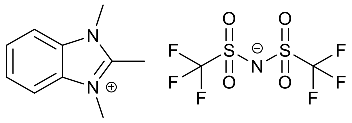

[tmbim][I] (8.4 mmol, 2.41 g) was dissolved in DCM (40 mL) and LiNTf$_2$ (9 mmol, 2.58 g) was dissolved in H_2O. The solutions were combined in a separating funnel, shaken for 10 minutes, and the organic phase collected and washed with H_2O (3 x 40 mL). The aqueous phase was extracted with DCM (3 x 40 mL), all organics phases combined, dried over $MgSO_4$, and then evaporated to dryness. The yellowish solid was recrystallised from hot ethanol and precipitated by cooling to 0 °C, resulting in a yellow solution and white crystals. The solids were recovered by cold filtration, washed with cold H_2O and recrystallised twice more. Yield 1.7 g, 3.85 mmol, 48%.

^{1}H NMR (400 MHz $CDCl_3$): δ = 7.62 (m, 4H, 4H$_{arom}$), 3.97 (s, 6H, [NCH$_3$]$_2$), 2.88 (s, 3H, [CH$_3$]).

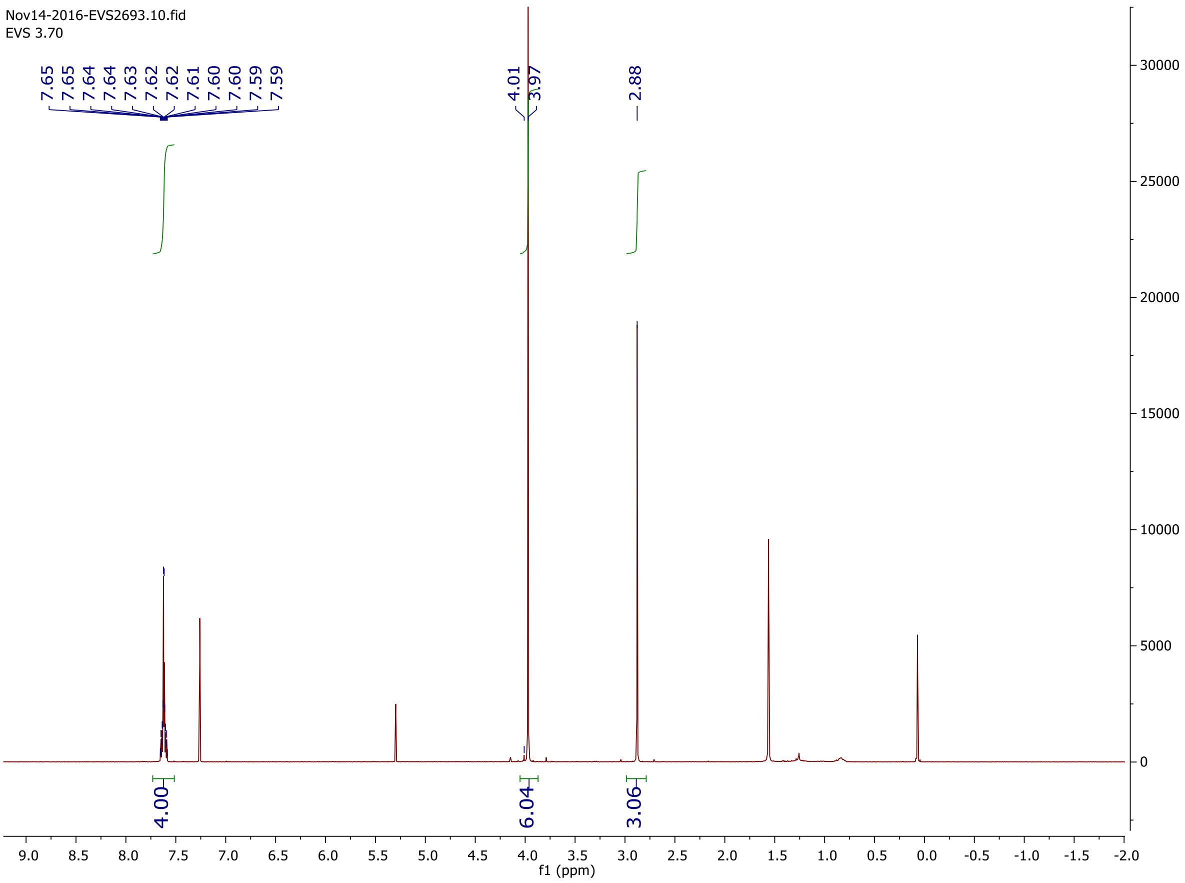

Nov14-2016-EVS2693.10.fid
EVS 3.70
7.65 7.65 7.64 7.64 7.63 7.62 7.62 7.61 7.60 7.60 7.59 7.59
4.01
3.97
2.88
4.00
6.04
3.06
f1 (ppm)

2. Electrochemistry

2.1 Aqueous CVs of precatalysts 1-7

1 dimethyl-pyalk

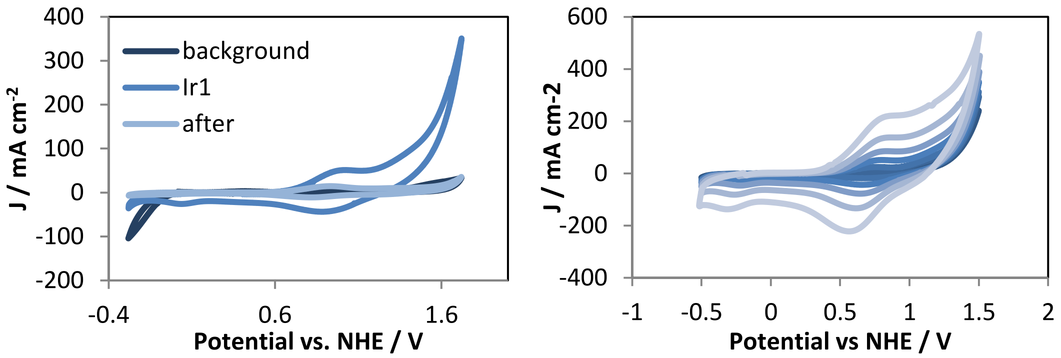

2 diphenyl-pyalk

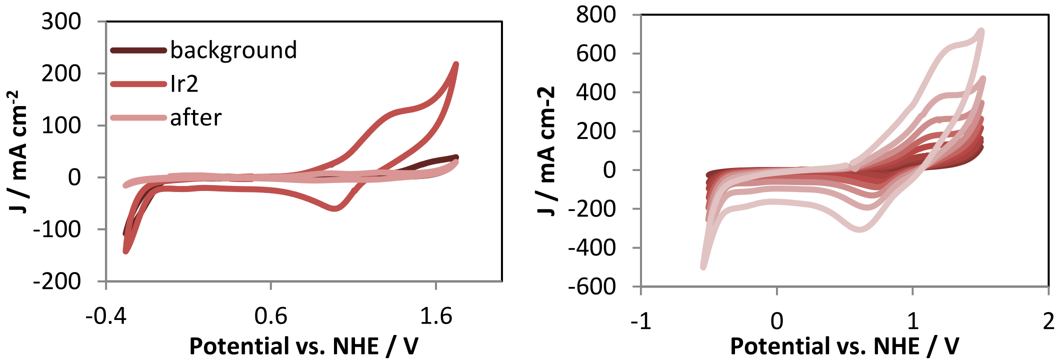

3 ditbutyl-pyalk

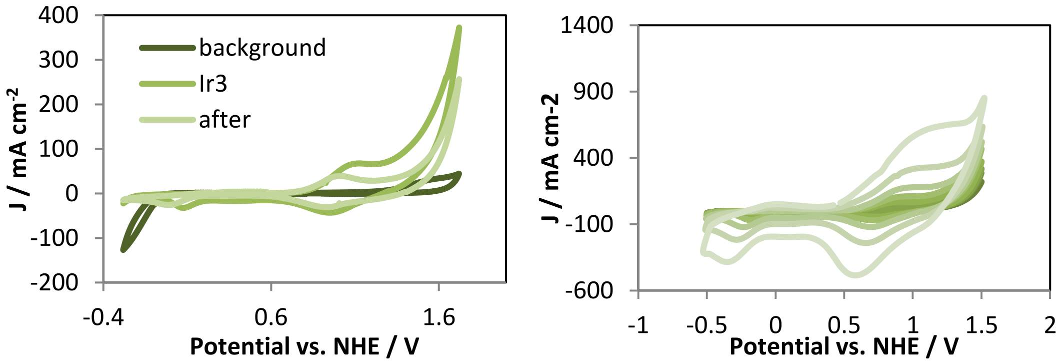

4 cyclohexyl-pyalk

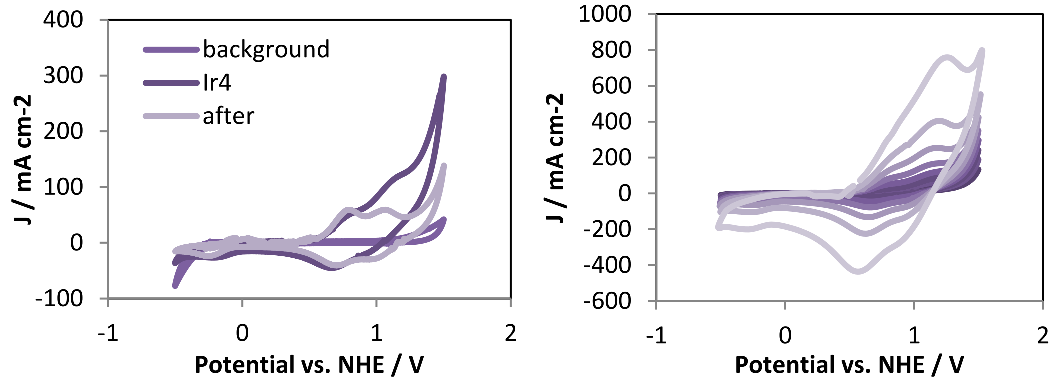

5 dimethyl-quinalk

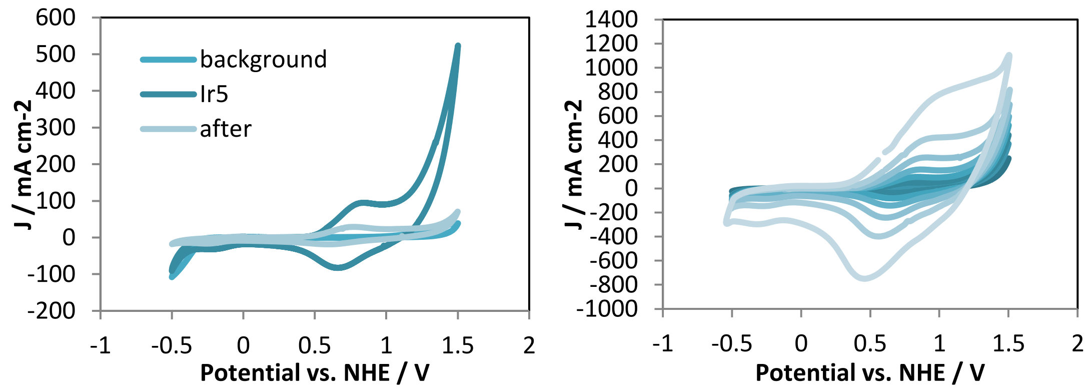

6: tetramethylcyclohexyl-pyalk

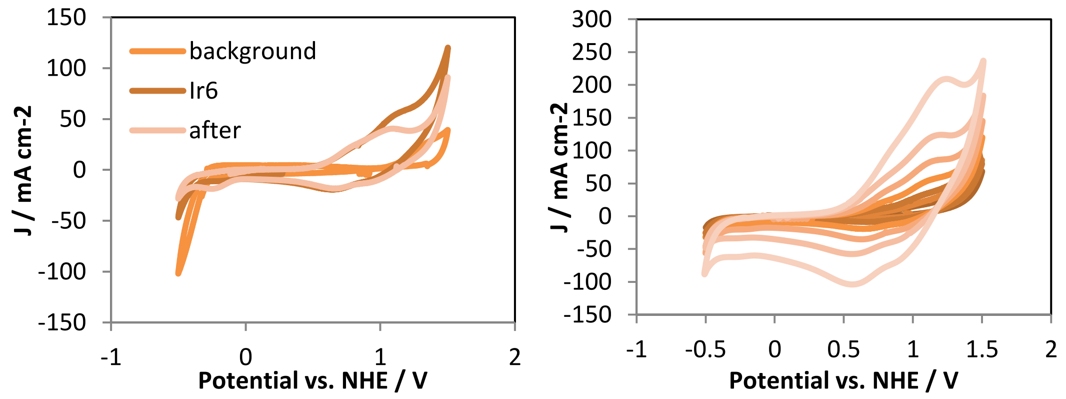

7: diphenyl-quinalk

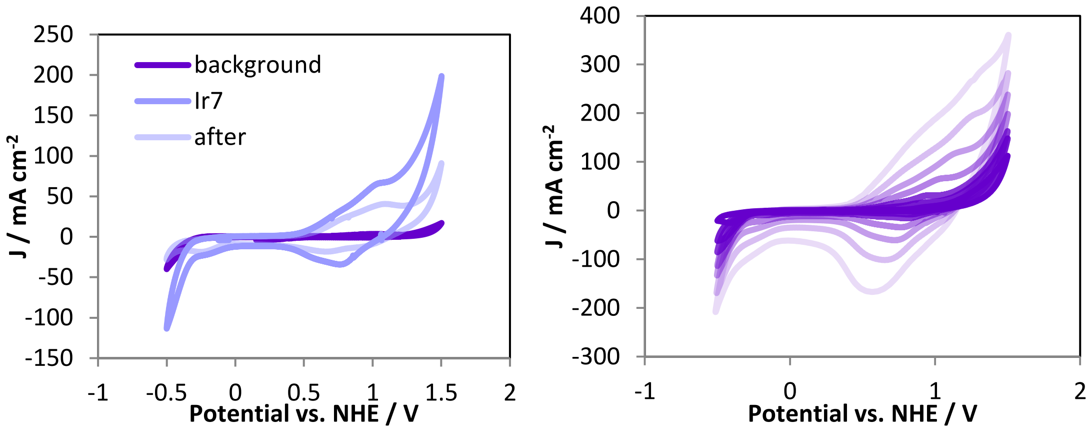

Figure S1. CVs of precatalysts **1-7** in 0.1M $NaNO_3$ in H_2O/tBuOH 4:1 with 1cm^2 FTO working electrode, Ag/AgCl reference electrode, Pt wire counter electrode. Left hand trace shows deposition on to the electrode, showing the response when a clean electrode is used in electrolyte (background), then with the precatalyst (Ir) and then the same electrode in a fresh solution of just electrolyte (after) with ν = 250 mVs^{-1}. Right hand trace shows scan rate dependence, with scan rate measured at 10 (dark), 50, 100, 250, 500, 1000 and 2000 (light) mVs^{-1}

1.2 Non-Aqueous CVs of ferrocene and precatalysts 1-7

1.2.1 Fresh electrolyte solution after each catalyst run

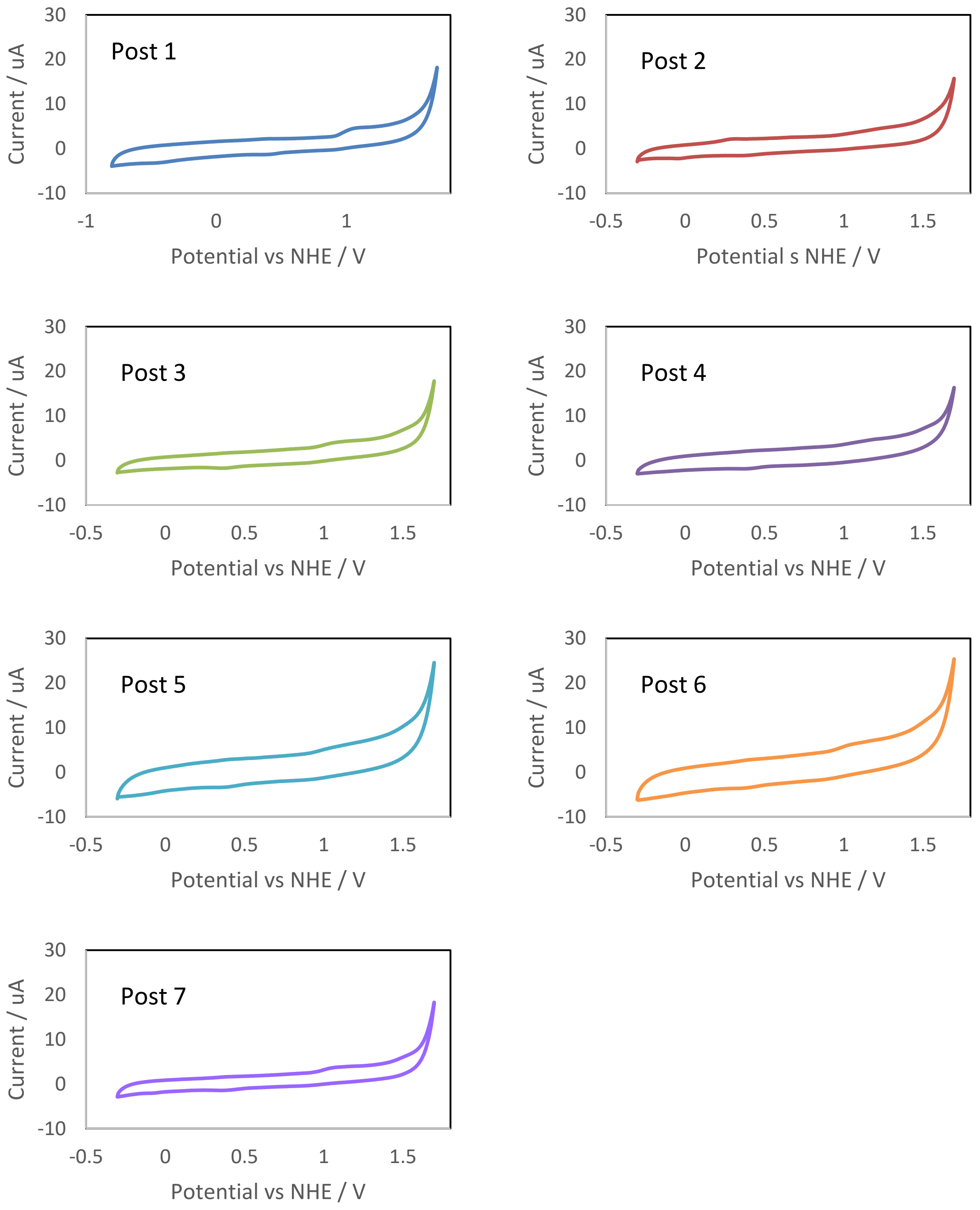

Figure S2. CVs of fresh electrolyte after each catalyst run showing no deposition was observed in DCM. Glove box conditions. 10mM [Ir], 0.15 M electrolyte, DCM (degassed, freeze pump thawed), glassy carbon working electrode, diameter 0.3 cm, $Ag/AgNO_3$ reference electrode, Pt wire counter electrode, 100 mVs^{-1} scan rate.

1.2.2 Scan rate dependence of non aqueous CVs for Ferrocene and precatalysts 1-7

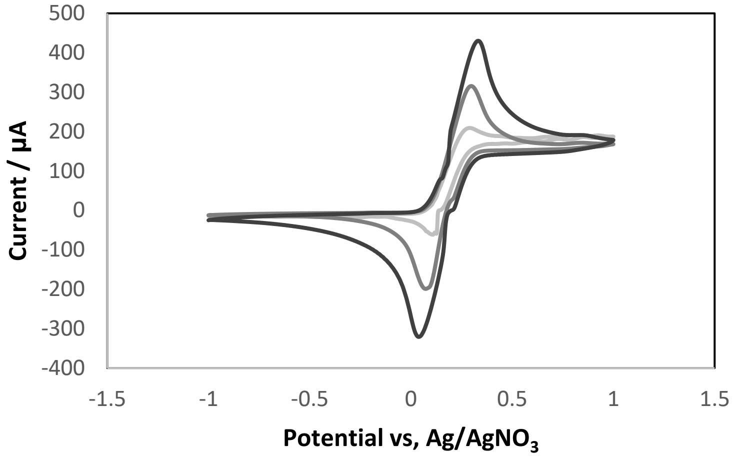

Ferrocene

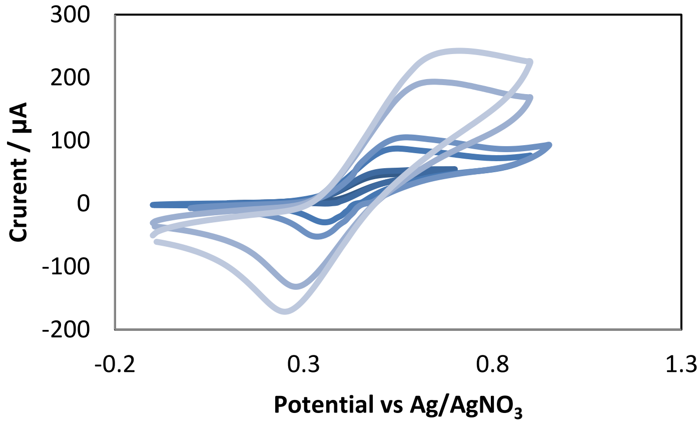

1

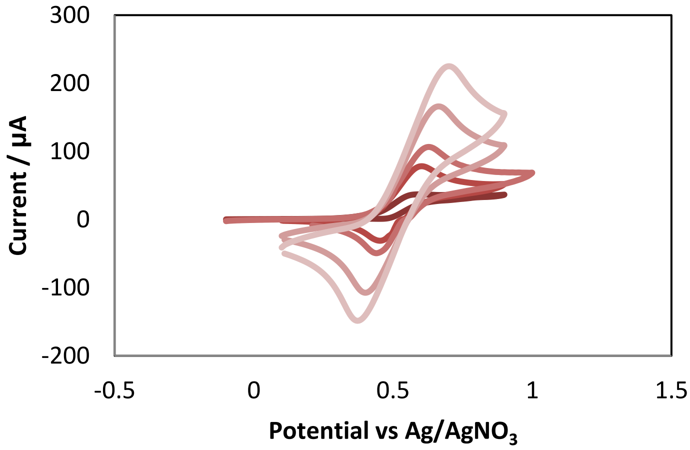

2

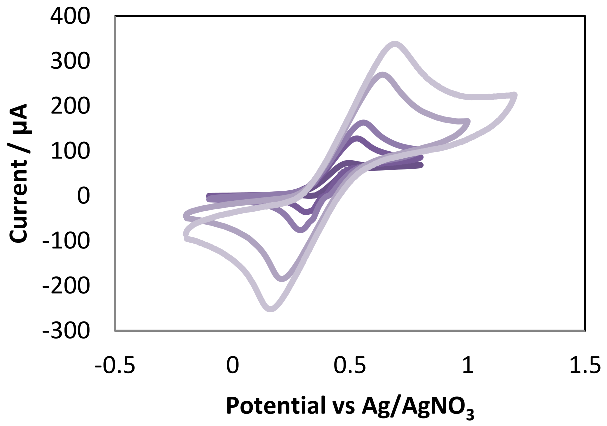
400
300
200
100
0
-100
-200
-300
Current / μA
-0.5
0
0.5
1
1.5
Potential vs Ag/AgNO3

4

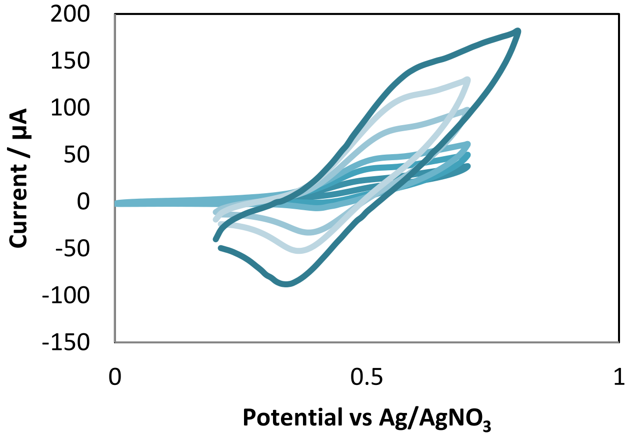
200
150
100
50
0
-50
-100
-150
Current / μA
0
0.5
1
Potential vs Ag/AgNO3

5

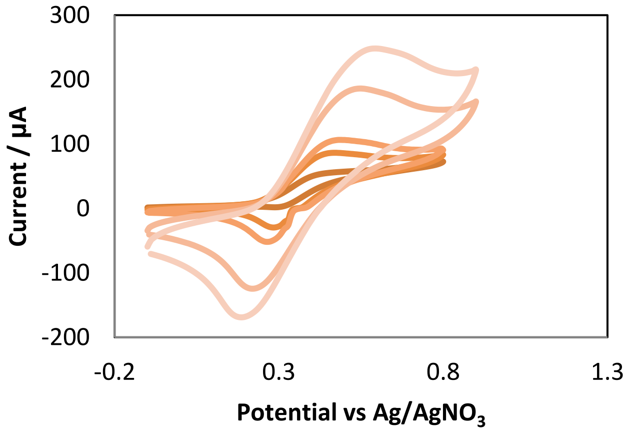
300
200
100
0
-100
-200
Current / μA
-0.2
0.3
0.8
1.3
Potential vs Ag/AgNO3

6

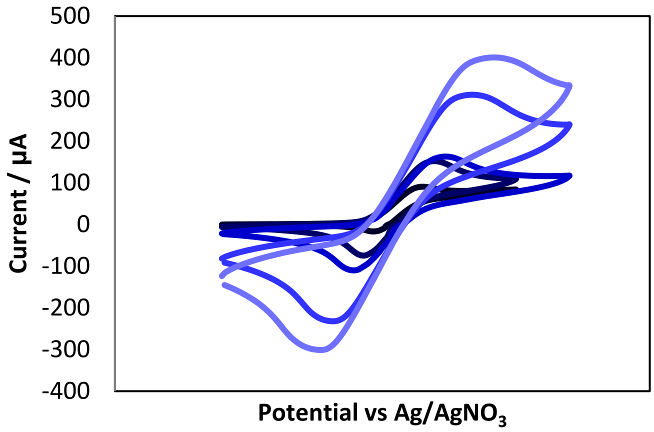

7

Figure S3. CVs of precatalysts **1-7** in 0.1M $NaNO_3$ in H_2O/tBuOH 4:1 with 1cm^2 FTO working electrode, Ag/AgCl reference electrode, Pt wire counter electrode. Scan rate increases with lighter colour from 5 (dark), 10, 50, 100, 500 to 1000 (light) mVs^{-1} for precatalsysts **1-7**. Ferrocene scan rates at 10, 50 and 100 mVs^{-1}

Table S1. Numerical data from CVs for precatalysts **1-7** from figure S2 at 100 mVs^{-1}

**at 100 mV s^{-1}**	**E_{ox} vs Ag/$AgNO_3$ / V**	**E_{red} vs Ag/$AgNO_3$ / V**	**ΔE / V**	**E_{mid} vs Ag/$AgNO_3$ / V**	**E_{mid} vs NHE / V**	**Approx. Peak i_{ox} / Peak i_{red}**
Ferrocene	0.365	0.015	0.35	0.19	0.36	-0.944
**1**	0.57	0.34	0.23	0.455	0.61	-1.11
**2**	0.63	0.44	0.19	0.535	0.69	-1.089
**3**	n/a	n/a	n/a	n/a	n/a	n/a
**4**	0.555	0.29	0.265	0.4225	0.58	-1.039
**5**	0.53	0.405	0.125	0.4675	0.63	-1.060
**6**	0.485	0.265	0.22	0.375	0.54	-1
**7**	0.735	0.395	0.34	0.565	0.72	-0.956
**1**[PF_6]	n/a	n/a	n/a	n/a	n/a	n/a

E_{ox} – potential of oxidation peak
E_{red} - potential of reduction peak
ΔE – peak to peak separation
Peak i_{ox} – peak current for oxidation
E_{mid} – mid point value of redox couple (E_{ox}-E_{red} / 2)

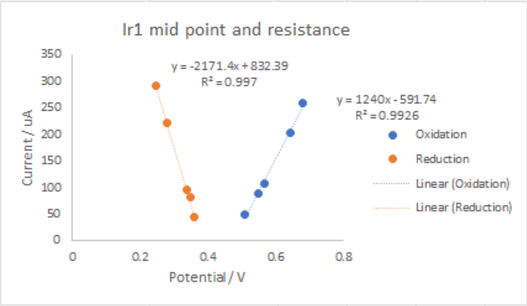
Ir1 mid point and resistance
y = -2171.4x + 832.39
R² = 0.997
y = 1240x - 591.74
R² = 0.9926
Oxidation
Reduction
Linear (Oxidation)
Linear (Reduction)
Current / uA
Potential / V

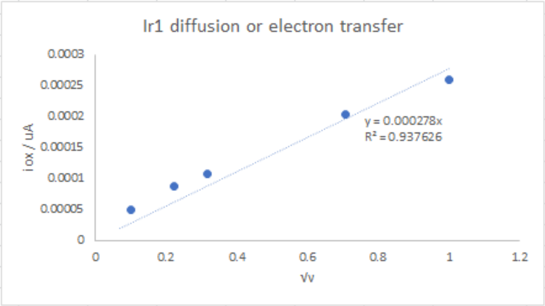
Ir1 diffusion or electron transfer
y = 0.000278x
R² = 0.937626
iox / uA
√v

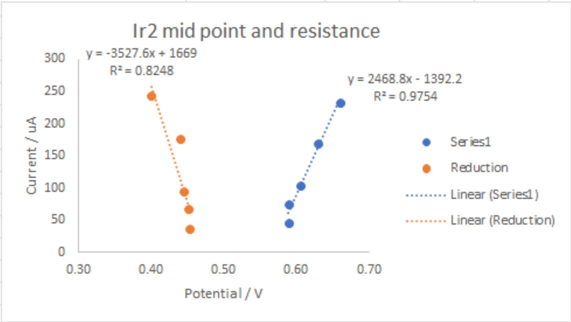
Ir2 mid point and resistance
y = -3527.6x + 1669
R² = 0.8248
y = 2468.8x - 1392.2
R² = 0.9754
Series1
Reduction
Linear (Series1)
Linear (Reduction)
Current / uA
Potential / V

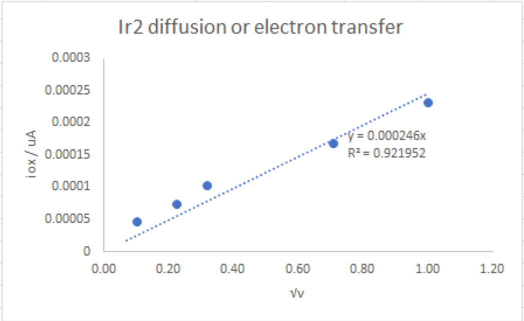
Ir2 diffusion or electron transfer
y = 0.000246x
R² = 0.921952
iox / uA
√v

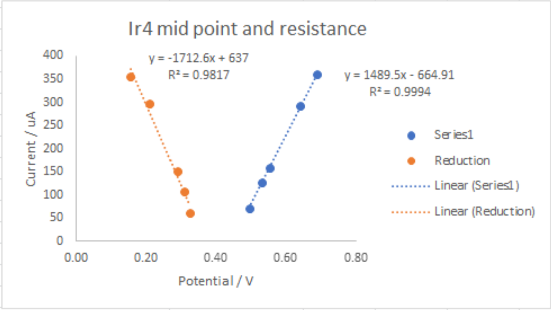
Ir4 mid point and resistance
y = -1712.6x + 637
R² = 0.9817
y = 1489.5x - 664.91
R² = 0.9994
Series1
Reduction
Linear (Series1)
Linear (Reduction)
Current / uA
Potential / V

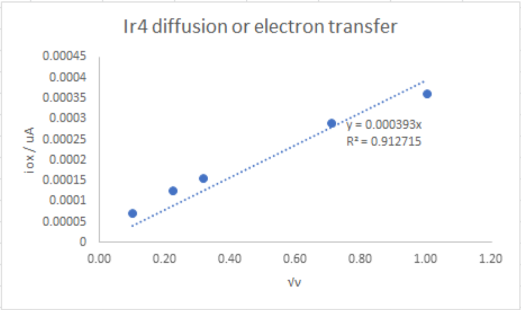
Ir4 diffusion or electron transfer
y = 0.000393x
R² = 0.912715
iox / uA
√v

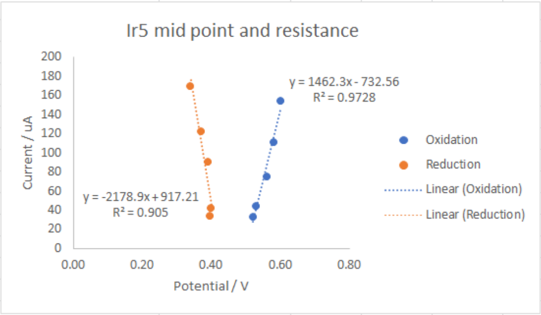
Ir5 mid point and resistance
y = 1462.3x - 732.56
R² = 0.9728
y = -2178.9x + 917.21
R² = 0.905
Oxidation
Reduction
Linear (Oxidation)
Linear (Reduction)
Current / uA
Potential / V

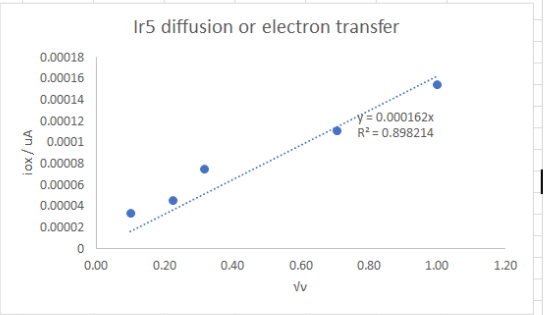
Ir5 diffusion or electron transfer
y = 0.000162x
R² = 0.898214
iox / uA
√v

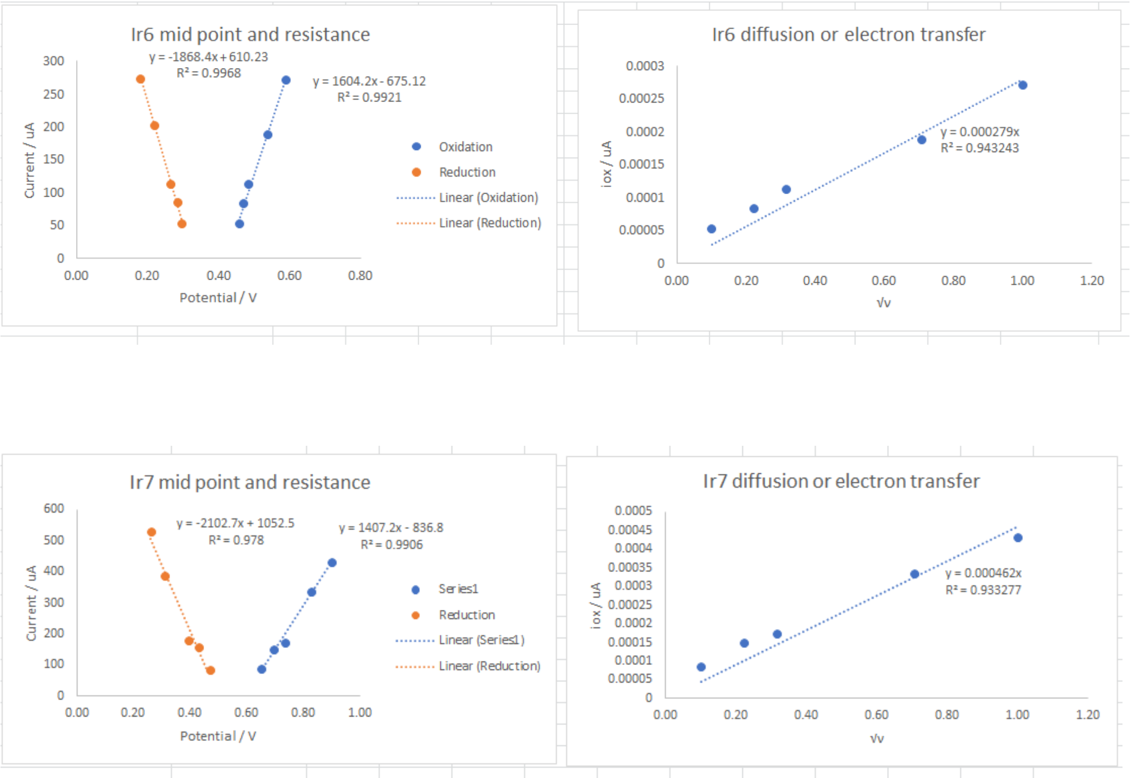

Figure S4. Mid point analyses and Randles-Sevick plots for **1-7** based on non-aqueous CV data.

3. Water Oxidation

2.1 Water oxidation with 20% tBuOH 100mM $NaIO_4$ 100µM [Ir]

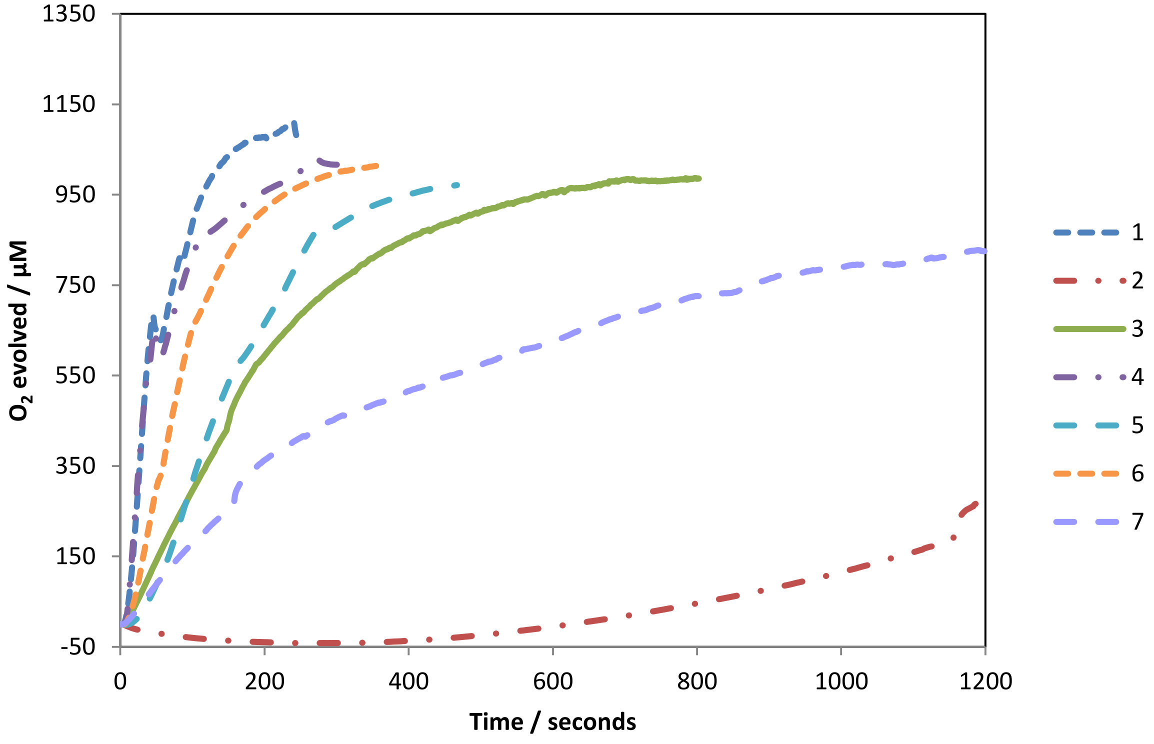

Figure S5. Oxygen evolution traces of precatalysts **1–7** at 100 µM [Ir] with 100 mM $NaIO_4$ in 4:1 $H_2O/^tBuOH$ (native pH 5.4) at 25 °C using a calibrated Clark electrode with stirring.

2.3 Water Oxidation with 100mM $NaIO_4$ 100µM [Ir] in D_2O

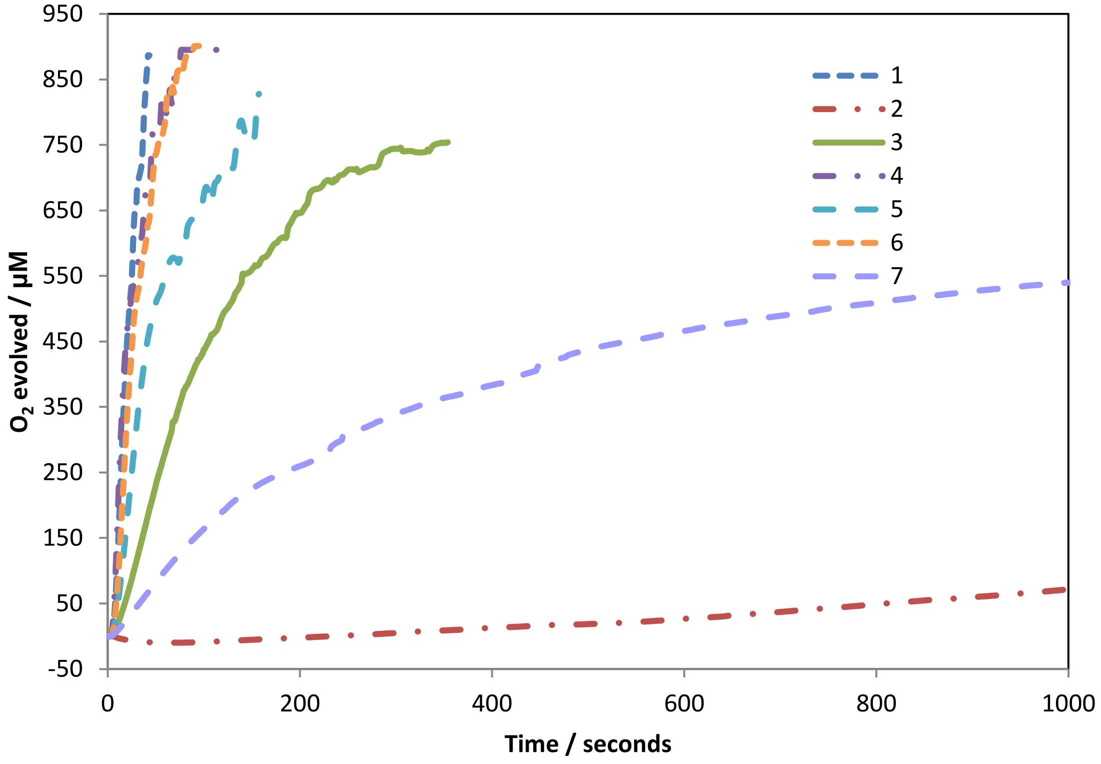

Figure S6. Oxygen evolution traces of the precatalysts **1–7** at 100 mM $NaIO_4$ in D_2O, 100 µM [Ir], 25 °C, using a calibrated Clark electrode with stirring.

2.6 Rate Averages

Table S2. Average initial rates for all water oxidation data from triplicate data

		k_{obs} $NaIO_4$ µM min^{-1}			k_{obs} CAN µM min^{-1}			k_{obs} tBuOH µM min^{-1}			k_{obs} D_2O µM min^{-1}		
	Repeats	4.115	4.619	4.512	1.794	1.718	2.016	1.251	1.267		2.260	1.998	2.147
1	Std Dev		0.053			0.126			0.008			0.107	
	Average		4.415			1.842			1.259			2.135	
	Repeats	0.020	0.018	0.019	0.194	0.292	0.179	0.015	0.018	0.020	0.007	0.008	0.006
2	Std Dev		0.001			0.050			0.002			0.001	
	Average		0.019			0.222			0.017			0.007	
	Repeats	0.437	0.403	0.407	0.515	0.415	0.408	0.145	0.127	0.188	0.336	0.312	0.331
3	Std Dev		0.015			0.049			0.025			0.010	
	Average		0.416			0.446			0.153			0.327	
	Repeats	3.628	3.590	3.914	1.632	1.987	2.055		1.220	1.272	2.126	1.893	1.877
4	Std Dev		0.019			0.186			0.026			0.114	
	Average		3.711			1.891			1.246			1.965	
	Repeats	1.309	1.175	1.207	1.292	1.163	1.197	0.204	0.159		0.680	0.743	0.827
5	Std Dev		0.057			0.055			0.022			0.061	
	Average		1.231			1.217			0.181			0.750	
	Repeats	2.842	2.905	2.901	1.226	1.072	1.075	0.834		0.987	1.769	1.479	1.452
6	Std Dev		0.029			0.072			0.076			0.143	
	Average		2.883			1.124			0.910			1.567	
	Repeats	0.142	0.146	0.128	0.086	0.090	0.094	0.135	0.122		0.101	0.103	0.125
7	Std Dev		0.008			0.004			0.007			0.010	
	Average		0.139			0.090			0.126			0.110	

2.6 Reaction Progress Kinetic Analysis by VTNA

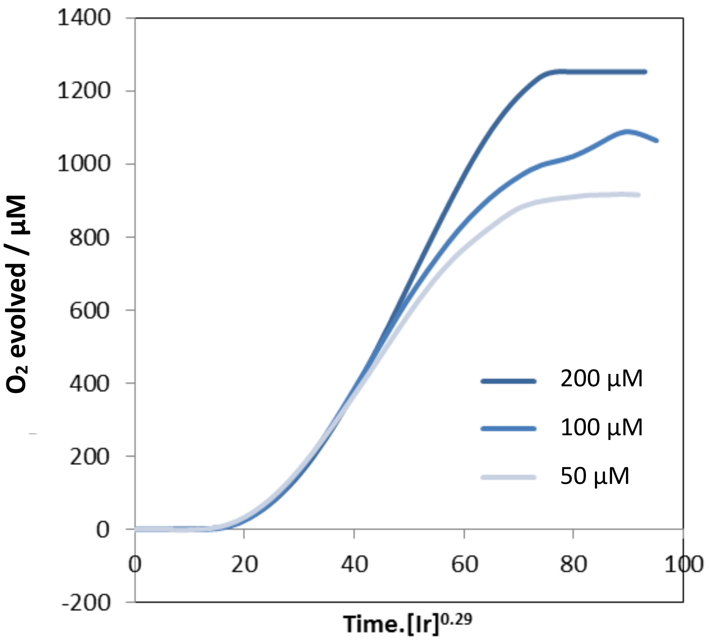

Figure S7. Oxygen evolution with VTNA applied at 100 mM $NaIO_4$ in H_2O, 200 µM, 100 µM, 50 µM of precatalyst **1** with best fit at 0.29 at native pH, 25 °C, using a calibrated Clark electrode

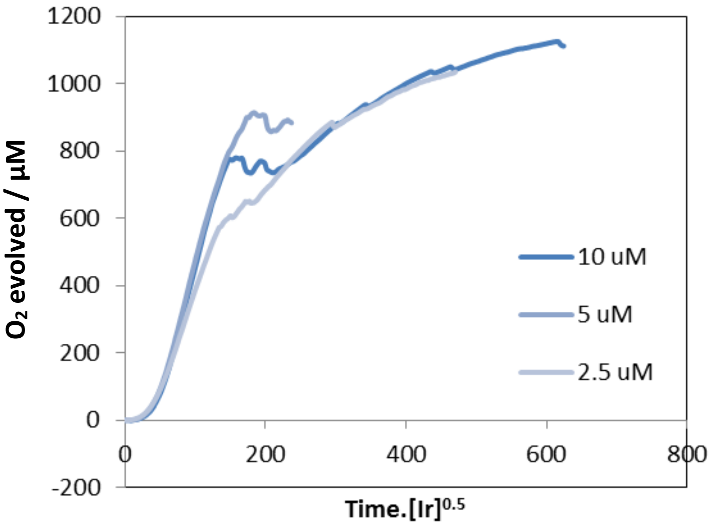

Figure S8. Oxygen evolution with VTNA applied at 10 mM $NaIO_4$ in H_2O, 10 µM, 5 µM, 2.5 µM of precatalyst **1** with best fit at 0.5 at native pH, 25 °C, using a calibrated Clark electrode

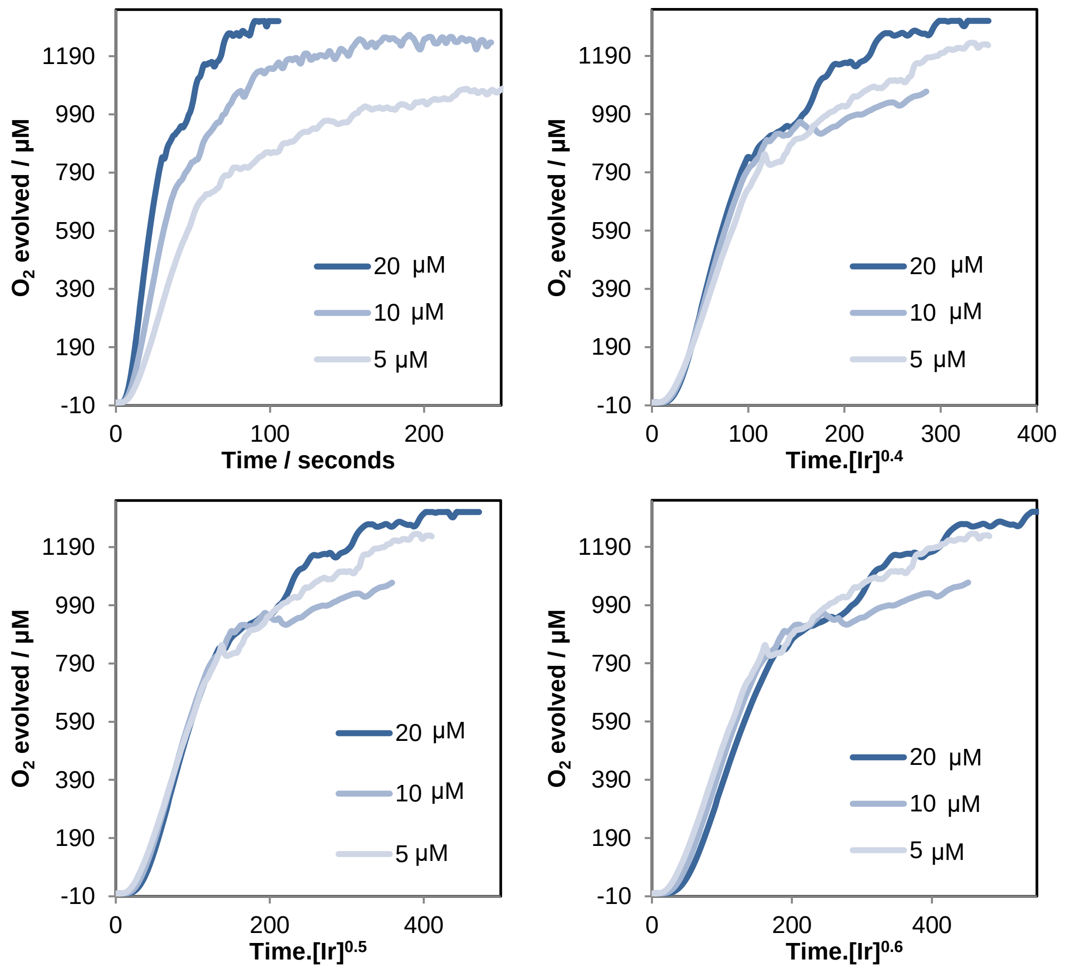

Figure S9 left) Oxygen evolution traces of precatalyst **1** at 100 mM $NaIO_4$ in H_2O, with varying [Ir] (20 µM, 10 µM, 5 µM), at 25 °C, using a calibrated Clark electrode; **right)** the same trace with VTNA applied to the x axis, where the factor has been iteratively changed to give the best overlap.

3 Electrochemically driven water oxidation

3.1 Cyclic Voltammograms of activated Iridium Catalysts

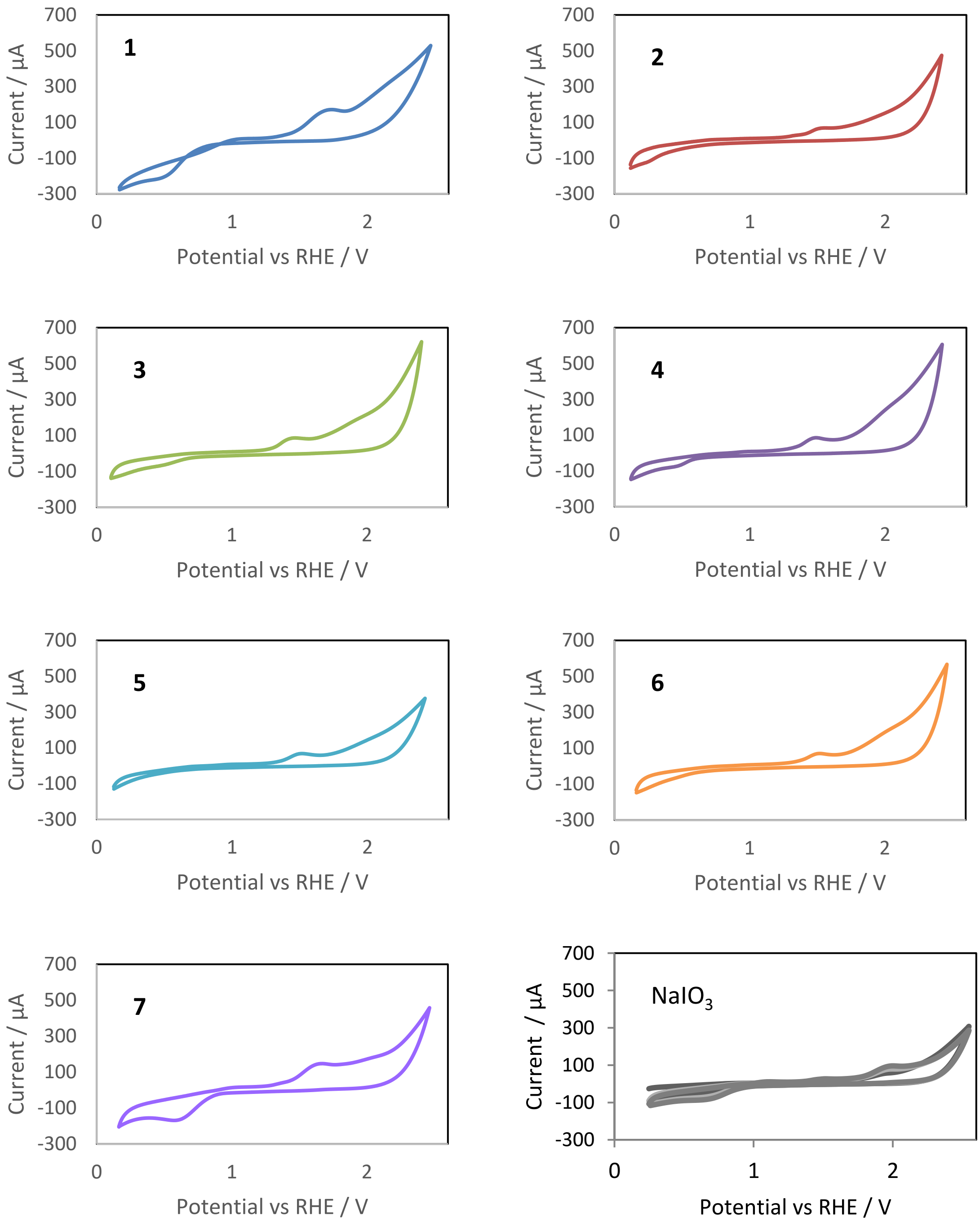

Figure S10. Cyclic voltammograms of complexes **1-7** preactivated with 50 equivalents of $NaIO_4$ (24 hours in H_2O at 1 mM [Ir]) at 1 mM [Ir] in H_2O with 0.1 M $NaNO_3$ at room temperature (WE: 3 mm glassy carbon disc, RE: Ag/AgCl, CE: 1 mm Pt wire, SR: 100 mVs^{-})

3.1 Electrochemical water oxidation set up

Initial testing with a three electrode set up inserted into the top of the Clark electrode chamber resulted in an overload of the Clark electrode. Within 10 seconds of immersion and before any potential had been applied, the Clark electrode reading showed a maximum value of oxygen evolution. Test experiments showed this response to originate from cross currents between the working electrode and the Clark electrode connected through the mains. Powering the potentiostat from the battery of a laptop effectively eliminated these cross currents.

A second potential problem was the possibility of hydrogen produced at the counter electrode to be registered by the Clark electrode, giving an inflated reading for the amount of oxygen evolved. As such the counter electrode (Pt wire) was encased in a glass sleeve such that any hydrogen produced would be kept separate from the rest of the solution and therefore shouldn't interfere with the oxygen measurement.

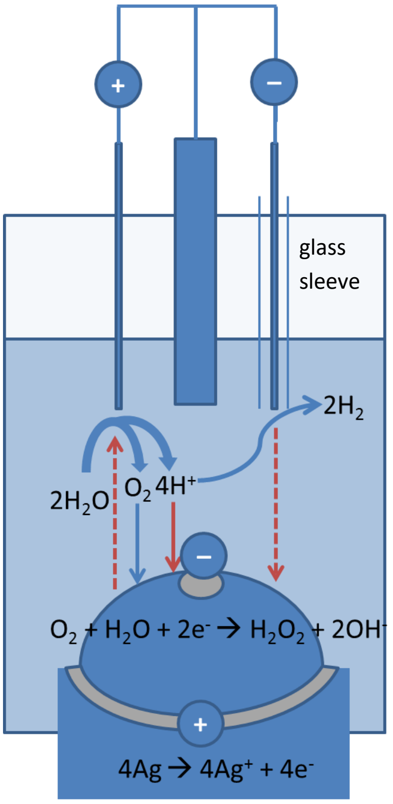

Figure S11. Schematic to represent the Clark electrode and potentiostat set up (not to scale).

3.2 Electrode optimisation

Several electrodes were investigated for use with the Clark electrode, with geometric fit into the chamber being a restriction. In all cases a Ag/AgCl refrence was used.

For Pt and Au mesh working electrodes, significant background water oxidation was observed at the desried potentials without any Ir catalysts added (figure S11 and S12).

Eventually a suitable electrode was found in a 0.5 × 0.5 cm BDD plate with electrical wire attached via epoxy resin sealed with silicone (figure S13).

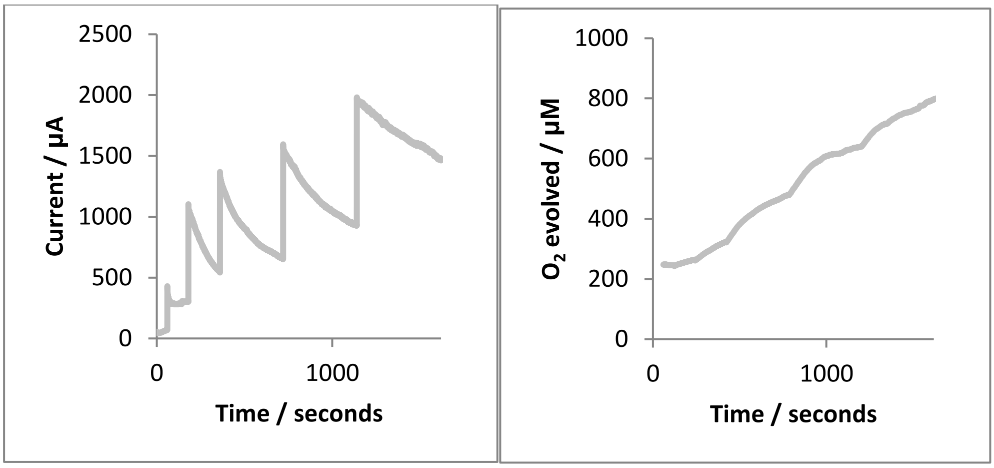

Figure S12. Chronoamperometry (left) and oxygen evolution (right) traces for a blank electrolyte solution (0.1 M $NaNO_3$) with a Pt mesh WE and a Pt wire CE. Chronoamperometry experiments were done at 1.4, 1.5, 1.6, 1.7, 1.8 and 1.9 V for 1,2,3,6,7,8 minutes respectively.

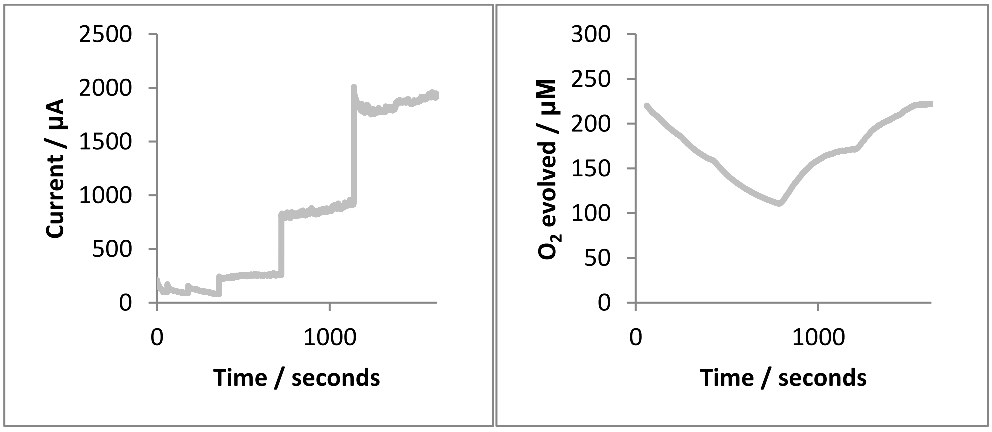

Figure S13. Chronoamperometry (left) and oxygen evolution (right) traces for a blank electrolyte solution (0.1 M $NaNO_3$) with a Au mesh WE and a Pt wire CE. Chronoamperometry experiments were done at 1.4, 1.5, 1.6, 1.7, 1.8 and 1.9 V for 1,2,3,6,7,8 minutes respectively.

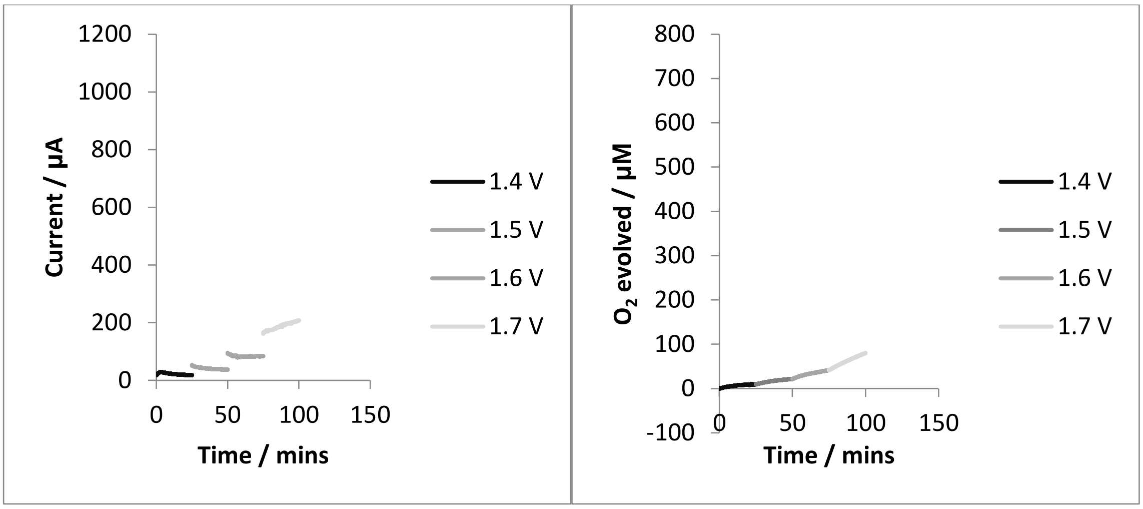

Figure S14. Chronoamperometry (left) and oxygen evolution (right) traces for a blank electrolyte solution (0.1 M $NaNO_3$) with a BDD plate WE and a Pt wire CE. Chronoamperometry experiments were done at 1.4, 1.5, 1.6 and 1.7 V for 25 minutes each.

3.4 Catalysts 1,3-7 electrochemcially driven water oxidation

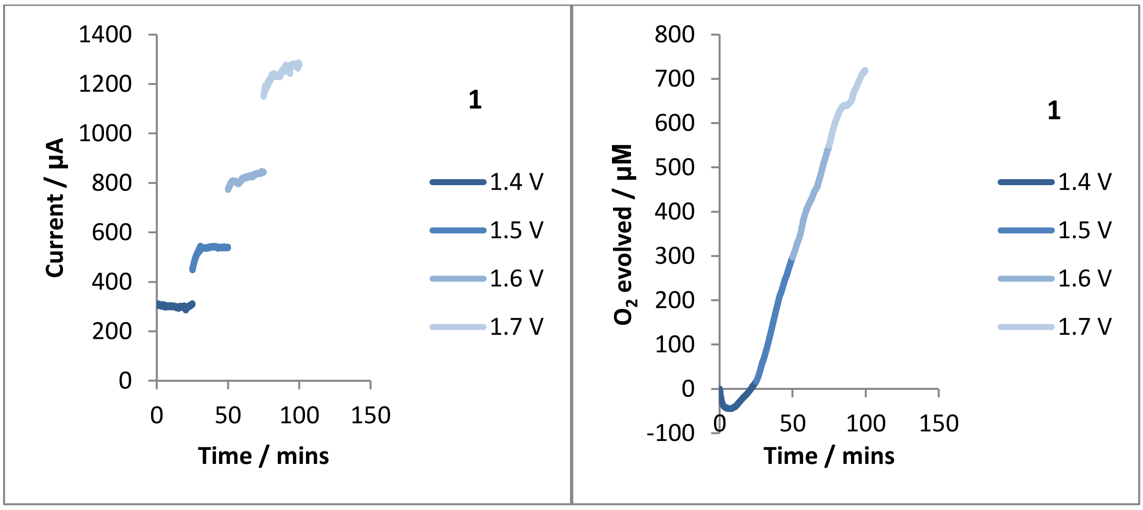

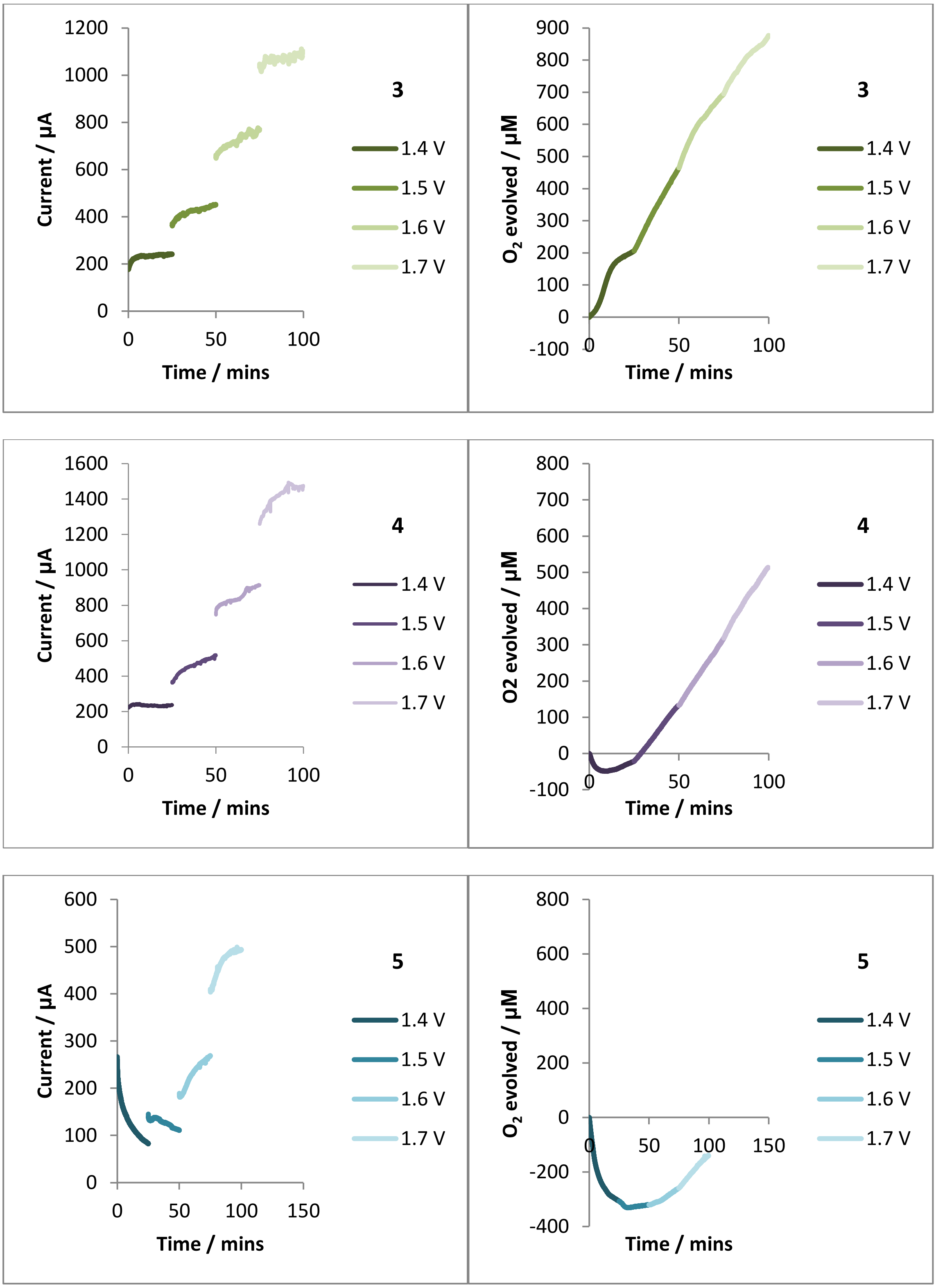

Current / µA
Time / mins
3
1.4 V
1.5 V
1.6 V
1.7 V
O_2 evolved / µM
Time / mins
3
1.4 V
1.5 V
1.6 V
1.7 V
Current / µA
Time / mins
4
1.4 V
1.5 V
1.6 V
1.7 V
O2 evolved / µM
Time / mins
4
1.4 V
1.5 V
1.6 V
1.7 V
Current / µA
Time / mins
5
1.4 V
1.5 V
1.6 V
1.7 V
O_2 evolved / µM
Time / mins
5
1.4 V
1.5 V
1.6 V
1.7 V

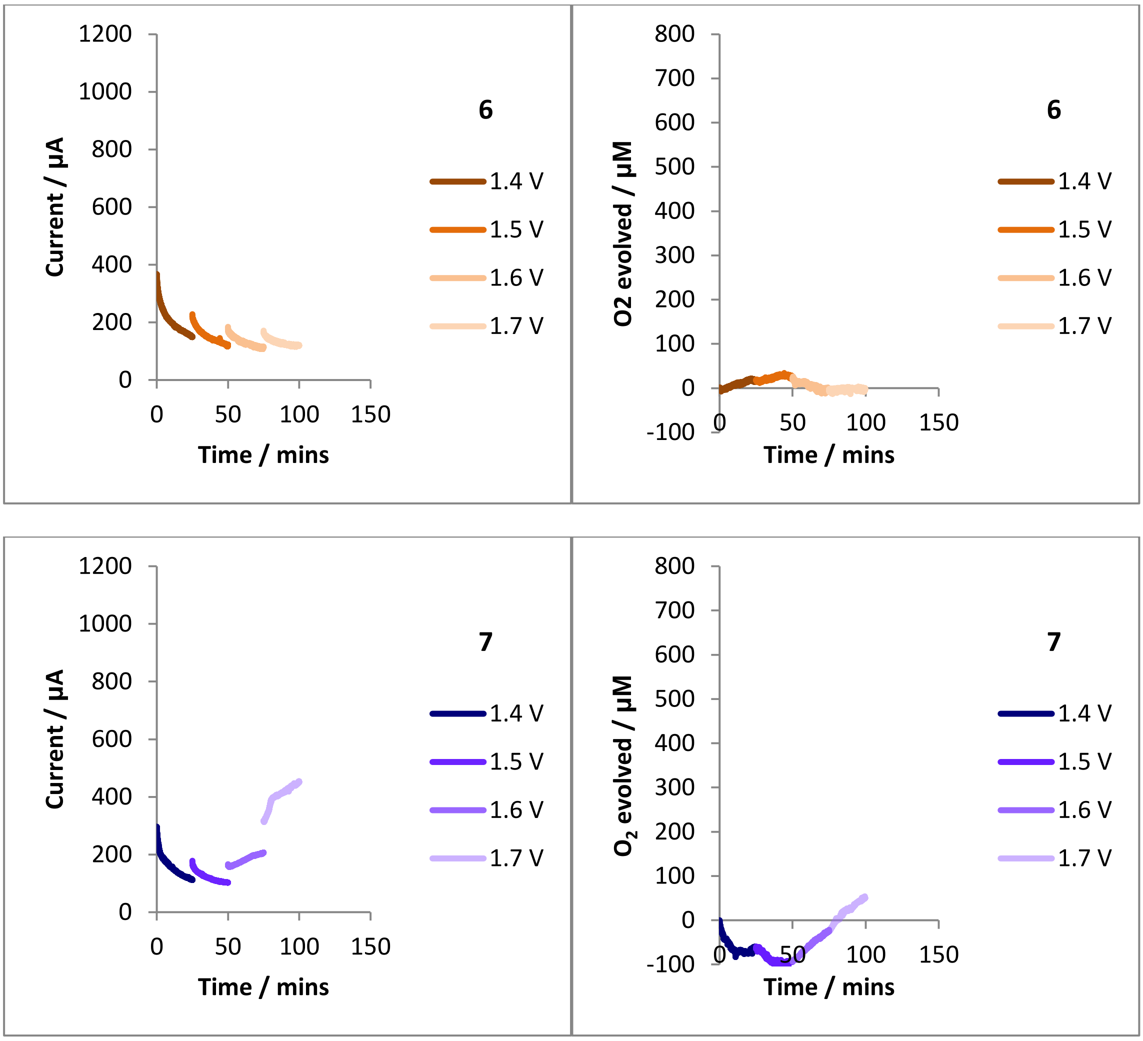

Figure S15. Chronoamperometry (left) and oxygen evolution (right) traces for electrochemically driven water oxidation using complexes **1**, and **3-7** chemically activated with 100 equivalents of $NaIO_4$ for 24 hours. All solutions were adjusted to a pH of ~6 with NaOH and HNO_3 WE: BDD plate $1cm^2$, CE: Pt wire, RE: Ag/AgCl, 2.5 mM [Ir1], 250mM $NaIO_3$. Chronoamperometry experiments were done for 25 mins at 1.4, 1.5, 1.6 and 1.7 V.

3.5 Blank electrolyte between catalyst runs

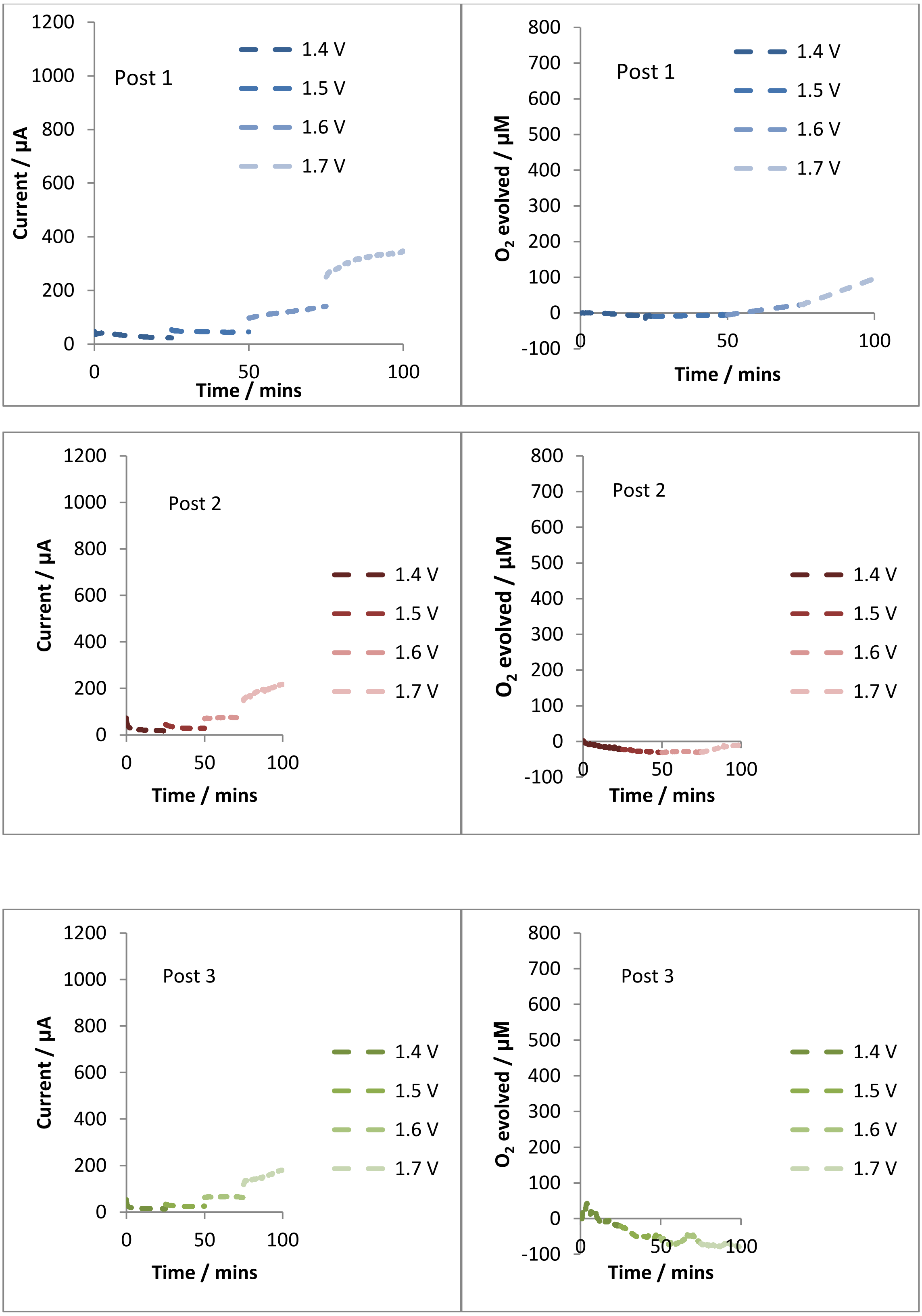

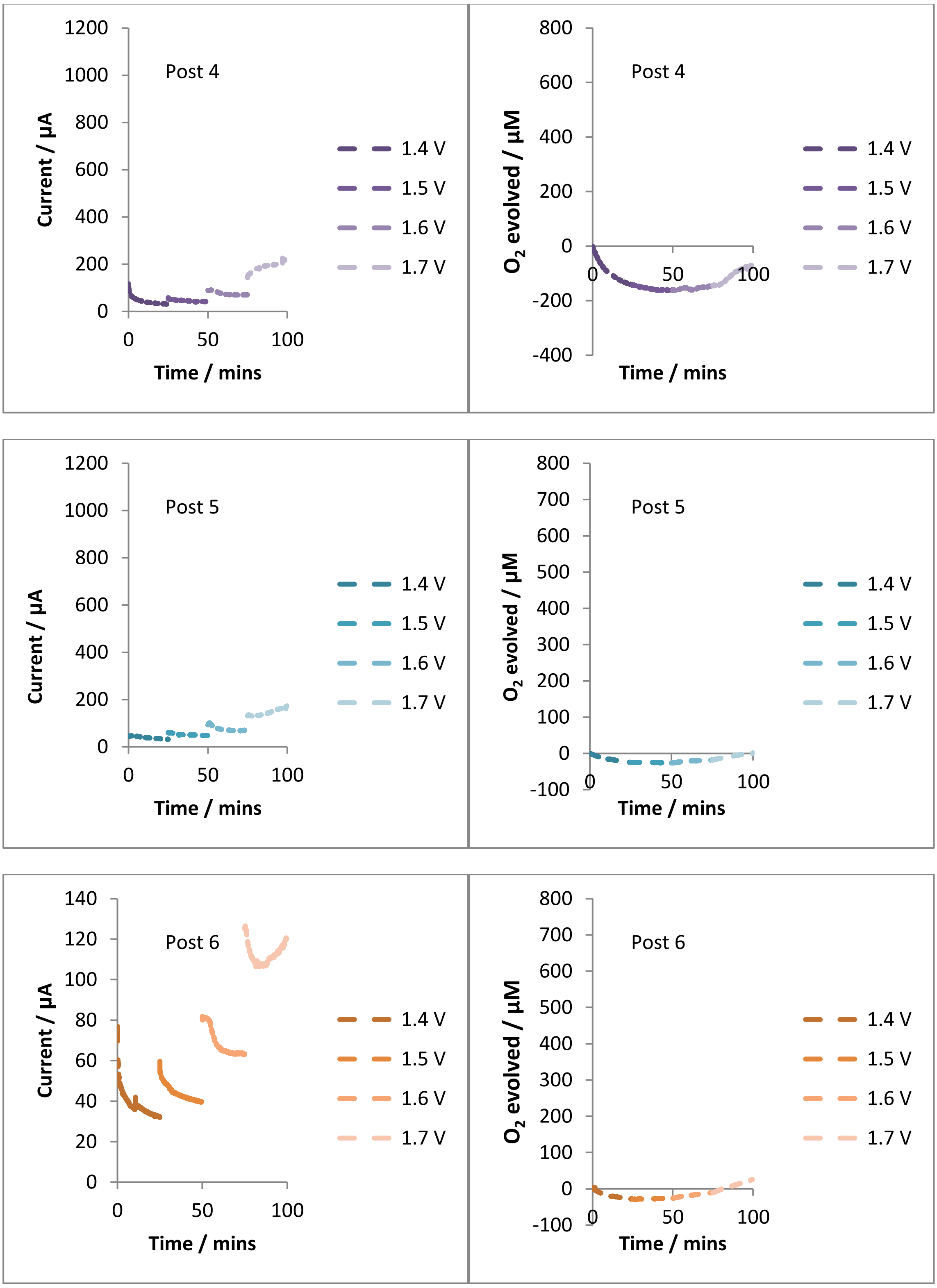
Post 4
Current / μA
1200
1000
800
600
400
200
0
0
50
100
Time / mins
1.4 V
1.5 V
1.6 V
1.7 V
Post 4
O2 evolved / μM
800
600
400
200
0
-200
-400
0
50
100
Time / mins
1.4 V
1.5 V
1.6 V
1.7 V
Post 5
Current / μA
1200
1000
800
600
400
200
0
0
50
100
Time / mins
1.4 V
1.5 V
1.6 V
1.7 V
Post 5
O2 evolved / μM
800
700
600
500
400
300
200
100
0
-100
0
50
100
Time / mins
1.4 V
1.5 V
1.6 V
1.7 V
Post 6
Current / μA
140
120
100
80
60
40
20
0
0
50
100
Time / mins
1.4 V
1.5 V
1.6 V
1.7 V
Post 6
O2 evolved / μM
800
700
600
500
400
300
200
100
0
-100
0
50
100
Time / mins
1.4 V
1.5 V
1.6 V
1.7 V

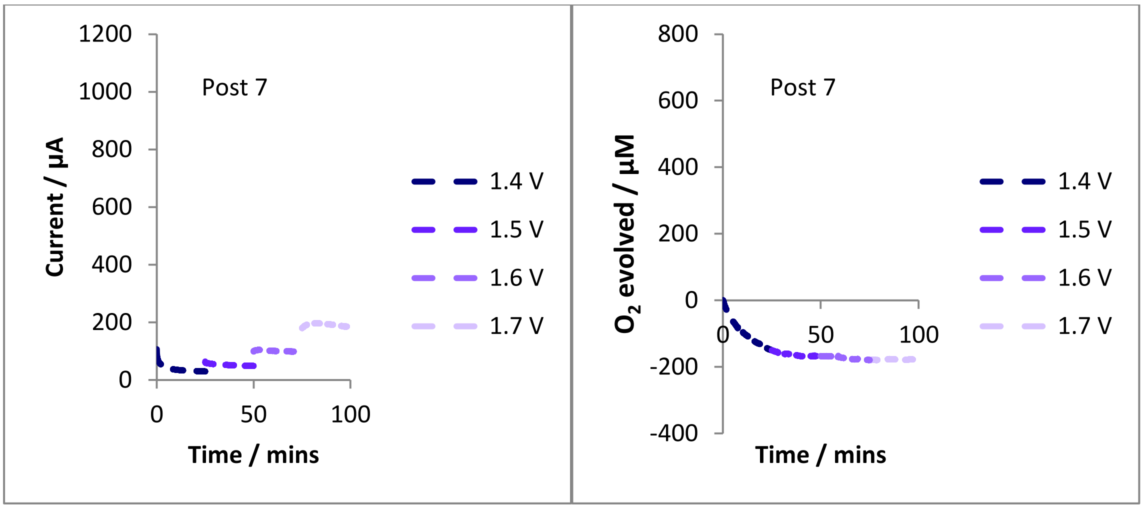

Figure S16. Chronoamperometry (left) and oxygen evolution (right) traces for electrochemically driven water oxidation in a solution of $NaIO_3$ (250 mM). All solutions were adjusted to a pH of ~6 with NaOH and HNO_3 WE: BDD plate 1cm^2, CE: Pt wire, RE: Ag/AgCl, 2.5 mM [Ir1], 250mM $NaIO_3$. Chronoamperometry experiments were done for 25 mins at 1.4, 1.5, 1.6 and 1.7 V.

References

1. W. Chen, J. Shen, T. Jurca, C. Peng, Y. Lin, Y. Wang, W. Shih, G. P. A. Yap, and T. Ong, 2015, 15207–15212.
